# Ferroptosis induces detrimental effects in chronic EAE and its implications for progressive MS

**DOI:** 10.1186/s40478-023-01617-7

**Published:** 2023-07-25

**Authors:** Priya Jhelum, Stephanie Zandee, Fari Ryan, Juan G. Zarruk, Bernhard Michalke, Vivek Venkataramani, Laura Curran, Wendy Klement, Alexandre Prat, Samuel David

**Affiliations:** 1 Centre for Research in Neuroscience and BRaIN Program, Research Institute of the McGill University Health Centre (RI-MUHC), Livingston Hall, Room L7-210, 1650 Cedar Ave., Montreal, QC H3G 1A4 Canada; 2 Neuroimmunology Research Laboratory, Centre de Recherche du Centre Hospitalier de l’Université de Montréal (CRCHUM), Montreal, QC H2X 0A9 Canada; 3 Department of Neuroscience, Faculty of Medicine, Université de Montréal, Montreal, Canada; 4 Research Unit Analytical BioGeoChemistry, Helmholz Zentrum München, German Research Center for Environmental Health, Neuherberg, Germany; 5 Comprehensive Cancer Center Mainfranken, University Hospital Würzburg, 97080 Würzburg, Germany

**Keywords:** Iron toxicity, Experimental autoimmune encephalomyelitis, Secondary progressive MS, Antioxidants, NCOA4, Lipid peroxidation

## Abstract

Ferroptosis is a form of lipid peroxidation-mediated cell death and damage triggered by excess iron and insufficiency in the glutathione antioxidant pathway. Oxidative stress is thought to play a crucial role in progressive forms of multiple sclerosis (MS) in which iron deposition occurs. In this study we assessed if ferroptosis plays a role in a chronic form of experimental autoimmune encephalomyelitis (CH-EAE), a mouse model used to study MS. Changes were detected in the mRNA levels of several ferroptosis genes in CH-EAE but not in relapsing–remitting EAE. At the protein level, expression of iron importers is increased in the earlier stages of CH-EAE (onset and peak). While expression of hemoxygenase-1, which mobilizes iron from heme, likely from phagocytosed material, is increased in macrophages at the peak and progressive stages. Excess iron in cells is stored safely in ferritin, which increases with disease progression. Harmful, redox active iron is released from ferritin when shuttled to autophagosomes by ‘nuclear receptor coactivator 4’ (NCOA4). NCOA4 expression increases at the peak and progressive stages of CH-EAE and accompanied by increase in redox active ferrous iron. These changes occur in parallel with reduction in the antioxidant pathway (system xCT, glutathione peroxidase 4 and glutathione), and accompanied by increased lipid peroxidation. Mice treated with a ferroptosis inhibitor for 2 weeks starting at the peak of CH-EAE paralysis, show significant improvements in function and pathology. Autopsy samples of tissue sections of secondary progressive MS (SPMS) showed NCOA4 expression in macrophages and oligodendrocytes along the rim of mixed active/inactive lesions, where ferritin+ and iron containing cells are located. Cells expressing NCOA4 express less ferritin, suggesting ferritin degradation and release of redox active iron, as indicated by increased lipid peroxidation. These data suggest that ferroptosis is likely to contribute to pathogenesis in CH-EAE and SPMS.

**Supplementary Information:**

The online version contains supplementary material available at 10.1186/s40478-023-01617-7.

## Introduction

There are currently limited effective treatment options available for primary progressive MS (PPMS) and secondary progressive MS (SPMS) [1–3]. Anti-inflammatory treatments that are effective in relapsing–remitting MS (RRMS) are not effective in progressive MS, suggesting that inflammation may not be the key driver of progressive MS pathology [1, 2, 4]. There is evidence that oxidative stress that leads to neurodegeneration may be an important contributor to progressive MS [4–6]. Recent studies have shown that in SPMS, chronic active lesions are surrounded by a rim of iron containing microglia/macrophages, called iron rim lesions (IRLs), which expand over time compared with non-IRLs which shrink [7–11]. These IRLs in SPMS are significantly more destructive than non-IRLs, form the leading edge of chronic inflammatory MS lesions and occur in the absence of signs of peripherally mediated inflammation [7, 8, 11–15]. Excess iron can induce neurodegeneration via several mechanisms [16].

We and others have shown iron deposition occurs in the spinal cord in experimental autoimmune encephalomyelitis (EAE), an animal model widely used to study MS [17–20]. Importantly, we showed that iron deposition is greater in chronic EAE (CH-EAE) than in relapsing–remitting EAE (RR-EAE) [20]. Iron deposition in CH-EAE is seen in the progressive stage of EAE (22–25 days after immunization, i.e., after the peak stage) and the late stage (2 months after immunization) [20]. In contrast, in RR-EAE, iron accumulation is detected at low levels in the late stage [20]. This suggests that iron may play a role in the later neurodegenerative changes in CH-EAE rather than in the early inflammatory demyelination stage and thus be of relevance for progressive MS. Iron is also required for remyelination [21, 22] and evidence from the marmoset EAE model suggests that at least some of the iron accumulation seen may contribute to lesion repair [17].

Iron is a redox-active metal that can produce free radicals. A regulated form of non-apoptotic cell death called ferroptosis was described about ten year ago in the context of cancer [23] but also in some neurodegenerative diseases including Parkinson’s disease and stroke [24–26]. Ferroptosis occurs when iron-mediated free radicals trigger lipid peroxidation in the absence of sufficient glutathione (GSH) mediated antioxidative defense [24, 25]. These changes result in the generation of damaging lipid radicals. Polyunsaturated fatty acids in membranes are highly susceptible to lipid peroxidation under oxidative conditions [27]. Greater damage of myelin lipids than myelin proteins is seen in the MS brain [28, 29]. Importantly, there is evidence that lipid peroxidation, iron toxicity, and glutathione deficiency occur in EAE and MS (discussed later). Recent studies have suggested a role for ferroptosis in oligodendrocyte loss in EAE [30] and in early T cell activation in EAE [31]. We have also recently reported that ferroptosis plays an important role in the rapid loss of oligodendrocytes and myelin in cuprizone-induced demyelination in the corpus callosum [32], an animal model also used widely in the MS field [33–35].

In the present study we first screened the mRNA expression of key ferroptosis markers in EAE and found that expression of these markers is altered in CH-EAE but not in RR-EAE. We then carried out detailed analyses at the protein level of the expression of these and other ferroptosis markers in CH-EAE that regulate intracellular iron (TfR1, DMT1, HO-1, ferritin and NCOA4), glutathione (GPX4 and xCT) and membrane lipid repair enzymes (ACSL4 and LPCAT3). Changes in the expression of these markers in CH-EAE are associated with increased lipid peroxidation and disease progression. The dysregulation of these molecules and the induction of lipid peroxidation in the later stages of CH-EAE is associated mainly with macrophages/microglia and oligodendrocytes in and near EAE lesions. Treatment with ferroptosis inhibitor (UAMC-3203) starting at the peak of the disease, significantly reduced disease severity, lesion burden and indicators of ferroptosis. These studies were extended to assess the expression of key ferroptosis markers in secondary progressive MS (SPMS) lesions in autopsy tissues. Together, these findings support a role for ferroptosis in CH-EAE and SPMS.

## Materials and methods

### Generation of experimental autoimmune encephalomyelitis, treatment with ferroptosis inhibitor and collection of tissues

RR-EAE and CH-EAE were induced as we have reported previously [36]. Female C57BL/6 mice (Charles River Laboratories, QC, Canada), 8–10 weeks of age were used for all experiments. RR-EAE was induced by subcutaneous injections of 50 μg MOG_35–55_ (MEVGWYRSPFSRVVHLYRNGK; Alpha Diagnostics Intl. Inc.) peptide emulsified in complete Freund's adjuvant (CFA) containing 0.5–1.0 mg/mL *Mycobacterium tuberculosis* (Fisher Scientific, Canada). 50 µl of this emulsion was injected on each side of the base of the tail. An intravenous injection of 200 ng pertussis toxin (Enzo life sciences, ML-G100-0050) was given at the time of the immunization and then repeated 48 h later. This protocol induces a monophasic or multiphasic remitting EAE disease course [36]. CH-EAE was induced by immunization with 300 μg MOG_35–55_ peptide in CFA containing 3–4 mg/mL *Mycobacterium tuberculosis*. Intravenous injections of 300 ng pertussis toxin were given at the time points mentioned above for RR-EAE. This protocol generates chronic EAE in which mice do not remit and remain at a high chronic disability score [36]. All procedures were approved by the McGill University Animal Care Committee and followed guidelines of the Canadian Council on Animal Care. Mice were evaluated daily, and clinical signs of EAE were graded as follows: Grade 0, normal; Grade 1, flaccid tail; Grade 2, mild hind-limb weakness (quick righting reflex); Grade 3, severe hind-limb weakness (slow righting reflex); Grade 4, hind-limb paralysis; and Grade 5, hind-limb paralysis and partial fore-limb weakness [20, 36, 37]. All animal work adhered to the ARRIVE guidelines [38]. Animals were allowed to acclimatize for 7 days before being used in experiments.

Mice were assigned randomly to treatment and control groups. The treatment group received the ferroptosis inhibitor UAMC-3203-HCL [39] (10 mg/kg body weight; HY-112909A-100MG, MedChem Express) intraperitoneally, daily for 15 days starting from the time they reached the peak stage of clinical paralysis (grade 3 or greater). Mice in the control group received vehicle (1% DMSO in normal saline) intraperitoneally for the same duration. The number of mice used for each group is indicated in Fig. 6 legend. The individual doing the clinical scoring was blinded to the treatment and control groups. Sample size was calculated based on our historical experience with EAE for medium effect size (f = 0.25), a confidence coefficient of 0.05 and a statistical power of 0.80.

Thoracic spinal cord tissue from CH-EAE mice was collected for immunofluorescence staining. The whole spinal cord was taken for q-PCR, Western blotting, CE-ICP-MS and Glutathione assays. Tissue was collected at the onset (grade 1; days 8–10), peak (grade 3–4; 12–15 days) stages of RR- and CH-EAE, and remission stage of RR-EAE (grade ≥ 2; days 18–22) or the progressive stage of CH-EAE (grade 3–4; 18–22 days) as described previously [20, 36] (Additional file 1: Fig. S1).

**Fig. 1:**
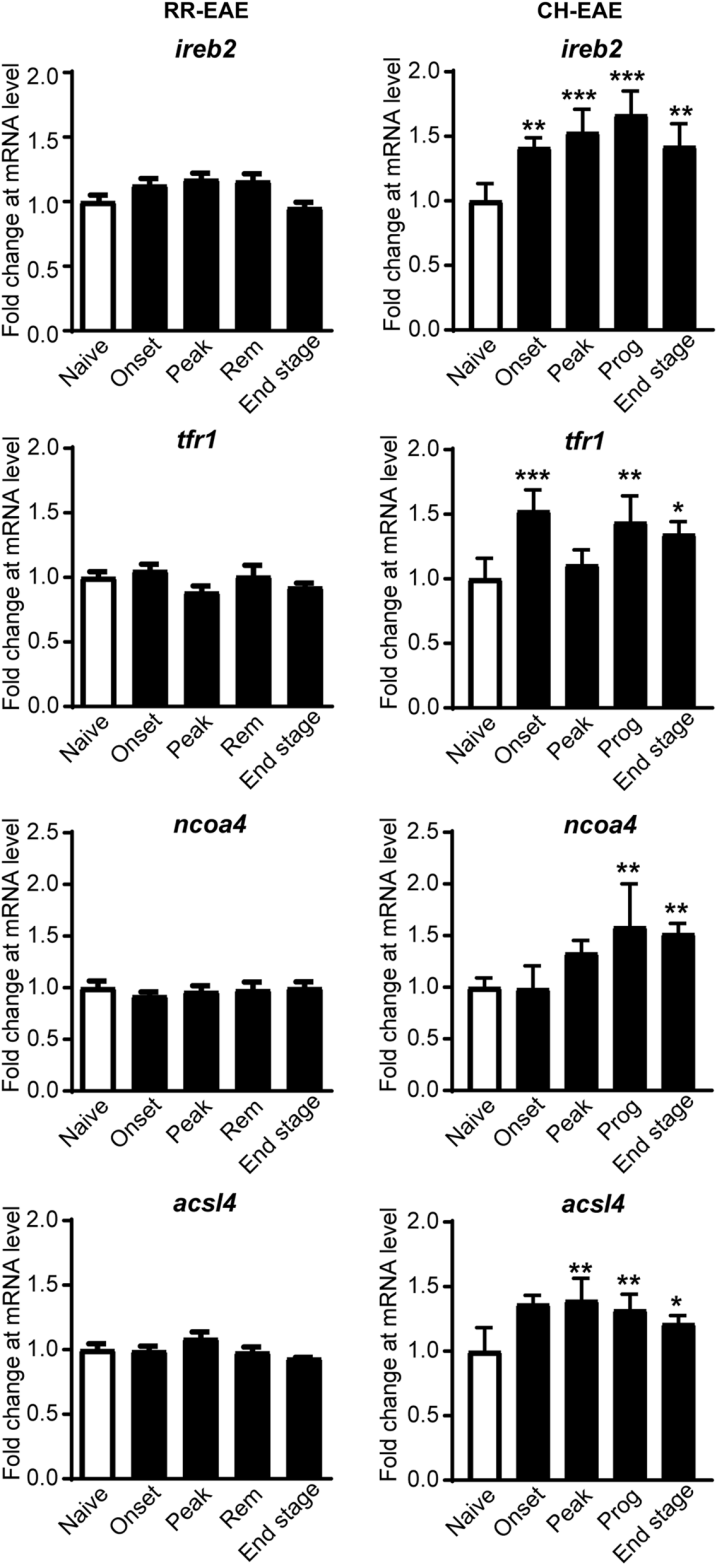
mRNA expression of select ferroptosis markers in CH-EAE and RR-EAE. Q-PCR screen of a select panel of ferroptosis markers at various stages of RR-EAE and CH-EAE. Note the significant increases in mRNA levels of *ireb2*, *tfr1*, *ncoa4* and *acsl4* in CH-EAE but not RR-EAE. (For CH-EAE (i) ireb2; naïve vs. onset and end stage: p = 0.003; naïve vs. peak and progressive stages: *p < 0.001; F*_*(4, 19)*_* = 12.54, p < 0.001* (ii) tfr1; naïve vs. onset, prog and end stage respectively: *p < 0.001,p = 0.003 and p = 0.02; F*_*(4, 19)*_* = 10.99, p < 0.001* (iii) ncoa4; naïve vs. prog and end stage respectively: *p = 0.004 and p = 0.007; F*_*(4, 19)*_* = 8.446, p < 0.001* (iv) acsl4; naïve vs. onset and peak stage respectively: *p = 0.002 and p = 0.001*; naïve vs. prog: *p = 0.01; F*_*(4, 19)*_* = 7.406, p < 0.001. n = 4–5* per group, one-way ANOVA with Tukey’s multiple comparison test. *p < 0.05, **p < 0.01 and ***p < 0.001

### Quantitative real time polymerase chain reaction (Q-PCR)

The whole spinal cord was collected after intracardiac perfusion with 0.1 M PBS from naïve and EAE mice at different stages of disease (n = 4–5 mice per group). Total RNA was extracted using RNeasy Mini Kit (Qiagen, Mississauga, ON) following manufacturer's instructions. cDNA was reverse transcribed using the Quantinova Kit (QIAGEN, Catalog #205411) and amplified in an ABI OneStep cycler (Applied Biosystems) with SYBR Green PCR Master Mix (Applied Biosystem) and primer pairs specific for the genes of interest as indicated in Additional file 5: Methods. Peptidylprolyl isomerase A was used as an internal control gene. The results were quantified using the ΔΔCT method following standardization relative to the internal control gene [40].

### Western blotting

After intracardiac perfusion with PBS, the whole spinal cord from C57BL/6 naïve mice (control group) and EAE mice at different stages of the disease (n = 4–5 mice per group) were dissected out and total protein extracted with 1% Nonidet P-40 (Sigma Millipore), 2% SDS, 2 mM EDTA, 1% sodium deoxycholate (BDH Chemicals), and 0.15 M sodium phosphate, pH 7.2, plus a protease inhibitor cocktail (Roche Diagnostics) as described previously [41]. Total protein concentration was estimated using Bio-Rad DC protein assay according to the manufacturer’s instructions (catalog #500-0121) and used for Western blotting. Details of methods and antibodies used are provided in Additional file 5: Methods.

### Immunofluorescence and confocal microscopy of mouse tissue

Mice were perfused via the heart with PBS, followed by 4% PFA in 0.1 M phosphate buffer. Spinal cords were removed, postfixed in the same fixative for 24 h, and cryoprotected in 30% sucrose and frozen for cryostat sectioning. Cryostat sections of the cervical, thoracic, and lumbar spinal cord (14 μm thick) were obtained on glass slides for immunofluorescence labeling. Details of the protocol and antibodies used are provided in Additional file 5: Methods. Tissue sections were viewed with a confocal laser scanning microscope (FluoView FV1000, Olympus) and micrographs taken with the FV10-ASW 3.0 software (Olympus) and processed using ImageJ software. Confocal images of 3–4 sections of the thoracic spinal cord (40×) were taken per animal (n = 4–5 mice per group) and the cells quantified from digital images using Image J software. Only CC1+ and CD11b+ cell bodies with DAPI labeled nuclei located in the parenchyma of the ventromedial white matter of the spinal cord away from the edge of the tissue were counted. The total number of CD11b+ or CC1+ cells in a given area was counted, as well as the number co-expressing different markers, and the mean and percentage of positively labeled cells calculated. Graphs were plotted using GraphPad prism.

### Immunofluorescence and confocal microscopy of human tissue samples

Human brain tissue was obtained at autopsy from patients diagnosed with clinical and neuropathological MS according to the revised 2010 McDonald’s criteria [42]. Tissue samples were collected by the Neuroimmunology Research Laboratory, Centre de Recherche du Centre Hospitalier de l’Université de Montréal (CRCHUM) with full ethics approval (BH07.001 and Nagano 20.332-YP) and informed consent as approved by the local ethics committee. Tissue staining was done at the RI-MUHC with ethics approval (Nagano FERIMS, and 2021-7219). Cryostat sections (7 µm thick) were picked up on glass slides, post-fixed with 4% PFA for 30 min at RT followed by antigen retrieval using Citrate buffer (pH6) for 1 h followed by PBS washing. Immunofluorescence staining protocol was similar to that detailed above. MS lesions were classified using tissue sections stained with Luxol fast blue/Haematoxylin and Eosin as well as Oil Red O staining [43] using the lesion classification described by Kuhlmann et al. [10]. The immunofluorescence staining protocol, and antibodies used are provided in Additional file 5: Methods. Tissue sections were viewed with a confocal laser scanning microscope (FluoView FV1000, Olympus) and micrographs taken in a z-stack series with the FV10-ASW 3.0 software (Olympus). In total 11 lesions were assessed that include mixed active/inactive actively demyelinating (MAIAD) and mixed active/inactive and post-demyelinating (MAIAPD) lesions from a total of 5 SPMS cases. The two types of lesions were distinguished based on the histological staining as the MAIAD lesions show ORO staining, while the MAIAPD lesions do not show ORO staining. Both lesions show myelin loss as detected by loss of Luxol fast blue staining. Details about each case and the controls are given in Table 1. Confocal images were taken from 4 to 5 areas along the rim of the per lesions. Likewise, for the NAWM group, confocal images were taken of 4 to 5 areas from each NAWM region from 5 SPMS cases. In the control group, the same number of images were taken from a total of 4 Control cases. For statistical analysis, the average of the cell counts from the 4–5 images from each lesion was calculated and then used to plot the graph. For this the number of CD68+ or TPPP+ cells in a given area was counted from digital images using Image J, as well as the number co-expressing different markers. The means and percentages of positively labeled cells were calculated and graphs plotted using GraphPad prism.

**Table 1:** As indicated above, 11 lesions that include MAIAD and MAIAPD lesions were assessed from a total of 5 SPMS cases

Tissue identifier code	Age	Sex	Type of disease	EDSS	Duration of disease	Cause of death	Number of lesions analyzed
AB172	60	F	SPMS	8.5	28 years	Pneumonia	02
AB103	48	M	SPMS	8.5	6 years	Suicide	02
AB200	61	M	SPMS	9.5	26 years	Pneumonia	04
AB184	65	M	SPMS	9	15 years	Medical assistance in dying	01
AB259	48	F	SPMS	7.5	22 years	Medical assistance in dying	02
AB014	46	M	Control	–	No Neurological disease	Unknown	–
AB105	42	M	Control	–	Other neurological disease control (Epilepsy; Non epileptic tissue was used)	NA	–
AB146	30	F	Control	–	Other neurological disease control (Epilepsy; Non epileptic tissue was used)	NA	–
AB159#2	67	M	Control	–	No Neurological disease	Died in surgery	–

*NA* not applicable, tissue came from surgery

### Immunohistochemistry for 4HNE

Cryostat sections of the mouse spinal cord (14 µm thick) from 4% PFA perfusion-fixed mice were used for 3,3' diaminobenzidine (DAB) immunohistochemistry for 4-HNE as described previously with some minor modification [20]. Cross sections through the same thoracic region of the spinal cord from naïve, onset, peak and progressive stages were used. Details of immunostaining protocol are provided in Additional file 5: Methods. Sections were viewed using an Axioskop2 plus bright field microscope (Carl Zeiss) at 10× magnification, and images were captured using the Bioquant Life Sciences software. The quantification of the 4-HNE staining intensity was assessed using ImageJ software threshold plug-in, and the mean integrated density was calculated, and graphs plotted using GraphPad prism.

### Histological staining and quantification

To assess immune cell infiltration and myelin loss, mouse spinal cord sections were stained with the Cresyl Violet and Luxol fast blue (LFB), respectively. For Cresyl Violet staining, sections were incubated in 0.1% Cresyl Violet solution followed by differentiation in ethanol and coverslipped with mounting solution. For LFB, sections were first dehydrated in 50–95% ethanol and incubated in a 0.1% LFB solution overnight at 37 °C. Slides were then allowed to cool at 4 °C and incubated in 0.05% lithium carbonate solution, followed by differentiation in ethanol and then coverslipped with Permount. Images of the thoracic spinal cord were taken using Axioskop2 plus microscope (Carl Zeiss) using the Bioquant image analysis software (Bioquant life Sciences). For each section, the total lesion area (area containing an immune cell infiltration per cross section of thoracic spinal cord) and area of myelin loss (visualized by a loss of LFB staining) was measured using ImageJ software and expressed as the percentage and graphs plotted using GraphPad prism.

### Capillary electrophoresis- inductively coupled plasma-mass spectrometry (CE-ICP-MS)

Spinal cord from normal (naïve) and EAE mice at the peak (clinical grade 3 or higher; 12–15 days post-immunization), and progressive stage (22–25 days post-immunization) were collected and lysed in modified RIPA lysis buffer containing 1 × PBS pH 7.4, 0.5% sodium deoxycholate, 1% NP-40 on ice using a sonicator. Cell lysates were centrifuged at 14,000 RPM for 20 min, and one aliquot of supernatants were sent on dry ice to Helmholtz Center Munich, Germany. The other aliquot was used for protein assay using the Bio-Rad DC kit (Cat. No 500-0121) and to estimate the amount of ferritin using the mouse ferritin ELISA kit from Abcam (ab157713). Speciation and quantification of Fe^2+^, Fe^3+^, and total iron were performed by CE-ICP-MS as previously described [44, 45]. In brief, samples were analyzed on a “PrinCe 706” CE system equipped with an uncoated capillary (85 cm × 50 µm ID) and a laboratory-constructed CE-ICP-MS interface for element selective quantification of separated iron redox species at ICP-DRC-MS. Fe^2+^/Fe^3+^ separation and quantification were performed in 20 mM HCl-electrolyte at + 25 kV separation voltage and ^56^Fe isotope detection at ICP-DRC-MS. DRC technology with NH_3_ as reaction gas was employed for interference-free detection of the ^56^Fe isotope. For quality control total iron was additionally determined by ICP-sf-MS and values compared to the sum of iron species quantified by CE-ICP-DRC-MS. Data are presented as total iron levels, Fe^2+^/Fe^3+^ ratio, and ferritin-iron content.

### GSH assay

After intracardiac perfusion with PBS, the whole spinal cord from naïve and EAE mice at different stages of the disease, and inhibitor treatment groups (n = 5–6 mice per group) was removed for analysis. GSH levels were measured using the Glutathione assay kit from Cayman Chemicals (Ann Arbor, MI Cat# 703002) following manufacturer’s instructions. Briefly, tissues were homogenized using a sonicator in 50 mM MES buffer containing 1 mM EDTA, followed by centrifugation at 10,000*g* for 15 min at 4 °C. Supernatant were collected and one aliquot used for protein estimation (Bio-Rad DC kit; Cat. No #500-0121) and the other aliquot deproteinated using equal amount of metaphosphoric acid and centrifuged at 3000*g* for 3 min followed by mixing with 4 M triethanolamine. Samples, standard, assay buffer, co-factor, enzyme mixture and Ellman’s reagent was added to 96-well plates and incubated for 30 min. Absorbance of each sample and standard was measured at 412 nm using a micro plate reader. Total GSH levels were determined using a standard curve calculation based on manufacturer’s instructions and followed by normalization with the protein concentration.

### Blinding and randomization

EAE mice were randomly assigned to treatment and control groups. Also, all procedures, data collection and all statistical analysis were carried out with the investigator blinded to the treatment and control groups.

### Statistical analyses

Data are shown as Mean ± Standard Error of the Mean (SEM). Statistical tests and graphs were performed using GraphPad Prisms 9.4.1 software. Statistical analyses were performed by using Mann–Whitney-U test, one-way or two-way ANOVA with post hoc Tukey or Bonferroni test for multiple comparisons as indicated. Differences were considered significant at p < 0.05.

## Results

### Differences in mRNA expression of ferroptosis markers in CH and RR-EAE

We have reported previously that this form of CH-EAE is associated with greater lesion burden, myelin loss and axonal damage, and increased expression of chemokines/cytokines and immune cell infiltration as compared to RR-EAE [36]. We have also reported that earlier and greater accumulation of iron occurs in CH-EAE than in RR-EAE [20]. Iron detected by Inductively Coupled Plasma Mass Spectrometry (ICP-MS) showed accumulation in the spinal cord of CH-EAE mice in the progressive stage (which corresponds to the remission stage in RR-EAE; days 22–25 post-immunization) and in the late stage (~ 2 months post-immunization) [20]. In contrast, RR-EAE mice only showed significant increase in iron in the late stage 2 months after immunization [20]. These results were also confirmed by ferritin immunostaining and DAB-enhanced Turnbull's blue histochemistry for iron [20]. In the current work, we therefore compared the mRNA expression of several ferroptosis markers that regulate intracellular iron levels: iron response element binding protein 2 (*ireb2*), transferrin receptor 1 (*tfr1*), nuclear receptor coactivator 4 (*ncoa4*), ferroptosis-related lipid repair enzyme, acyl-CoA Synthetase Long Chain Family Member 4 (*acsl4*) and antioxidant enzyme glutathione peroxidase 4 (*gpx4*). These results show significant increase in expression of ferroptosis-inducing molecules (*ireb2*, *tfr1*, *ncoa4* and *acsl4*) in different stages of CH-EAE but not in RR-EAE (Fig. 1). Note differences in expression between RR-EAE and CH-EAE at the onset and peak stages even though the tissue was collected at the same onset and peak grades. Interestingly, *gpx4* required for glutathione-mediated antioxidant defense, which should be increased to provide protection, remains unchanged (data not shown) indicating an insufficient antioxidant response to prevent ferroptosis. This broad screen of key ferroptosis markers suggests that ferroptosis is likely to occur in CH-EAE rather than in RR-EAE. We chose to compare the remitting form of EAE with the chronic form in the same mouse strain (C57BL/6) to avoid differences in genetic background. It would, however, be of interest to confirm these results with RR-EAE induced in SJL mice with PLP [46].

### Changes in protein expression of key ferroptosis markers involved in increasing iron in CH-EAE

**Fig. 2:**
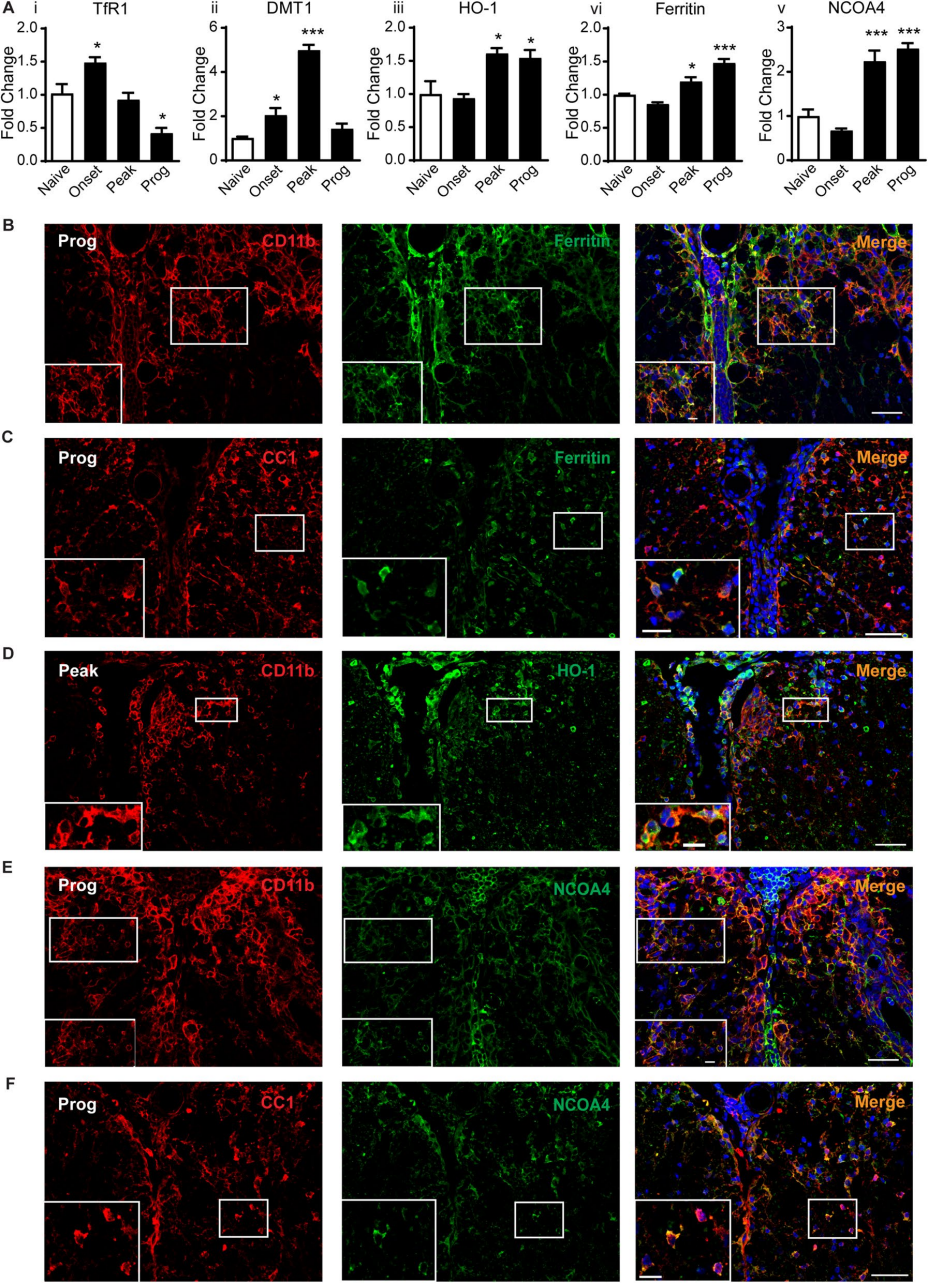
Changes in protein expression levels and cellular localization of iron-related ferroptosis markers. **A** Western blot data showing increased expression of TfR1 (onset), DMT1 (onset and peak), HO-1, ferritin, and NCOA4 (peak and progressive stages) of CH-EAE. (**A**i:TfR1; naïve vs. onset and prog: *p = 0.04 and p = 0.005* respectively; *F*_*(3, 16)*_ =  *17.21, p < 0.001*, **A**ii: DMT1; naïve vs. onset and peak: *p = 0.03 and p < 0.001* respectively; *F*_*(3, 15)*_ =  *57.40, p < 0.001*, **A**iii: HO-1; naïve vs. peak and prog: *p = 0.02 and p = 0.03* respectively; *F*_*(3, 16)*_* = 7.956, p = 0.002*, **A**iv: ferritin; naïve vs. peak and prog: *p = 0.04 and p < 0.001* respectively; *F*_*(3, 16)*_* = 31.42, p < 0.001*, **A**v: NCOA4; naïve vs. peak and prog: *p < 0.001* respectively; *F*_*(3, 15)*_* = 31.10, p < 0.001*. n = 4–5 per group, one-way ANOVA with Tukey’s multiple comparison test. *p < 0.05, **p < 0.01 and ***p < 0.001). **B**–**F** Double immunofluorescence labeling with rabbit anti-ferritin/rat anti-CD11b (**B**), rabbit anti-ferritin/mouse anti-CC1 (**C**), rabbit anti-HO-1/rat anti-CD11b (**D**), rabbit anti-NCOA4/rat anti-CD11b (**E**), and rabbit anti-NCOA4/mouse anti-CC1 (**F**). Areas in white rectangles are shown at higher magnification at the bottom of each panel. **B** Shows localization of ferritin in CD11b^+^ macrophages (53.1 ± 4.0%) and **C** in CC1^+^ oligodendrocytes (37.6 ± 3.9%) in the progressive stage of CH-EAE. **D** Shows localization of HO-1 in CD11b^+^ macrophages at the peak stage of CH-EAE (41.4 ± 2.68%). **E** Shows localization of NCOA4 in CD11b^+^ macrophages (49.6 ± 4.4%) (**E**), and **F** in CC1^+^ oligodendrocytes (42.8 ± 3.5%) **F** in the progressive stage of CH-EAE. Scale bars: 40 µm; Inset:12 µm

We next assessed changes in expression of iron-related ferroptosis markers at the protein level in CH-EAE by Western blot. This includes molecules that increase intracellular iron (TfR1, DMT1, HO-1, NCOA4 and ferritin) via different mechanisms [47]. The expression of TfR1, which imports transferrin-bound di-ferric iron into cells is increased early in the onset stage of EAE (Fig. 2Ai and Additional file 2: Fig. S2Ai), while expression of divalent metal ion transporter (DMT1), which imports divalent metals including Fe^2+^, is increased at the onset and even more at the peak of disease (Fig. 2Aii and Additional file 2: Fig. S2Aii), decreasing to pre-onset levels in the progressive stage. In contrast, the protein levels of HO-1, which extracts iron from heme found in heme-containing proteins that have been internalized by phagocytosis of damaged cells is significantly increased at the peak and progressive stages of CH-EAE (Fig. 2Aiii and Additional file 2: Fig. S2Aiii). Furthermore, as we reported earlier, the iron storage protein, ferritin, is steadily increased at the protein level in the peak and progressive stages of CH-EAE [20] (Fig. 2Aiv and Additional file 2: Fig. S2Aiv). Intracellular iron is stored in ferritin in its safe inactive ferric form [48]. The mobilization of redox active iron from ferritin is therefore a key factor in inducing ferroptosis [47]. The release of ferritin-bound iron is mediated by NCOA4, which binds ferritin and transports it to autophagosomes for degradation (ferritinophagy) and thus the release of bioactive iron [49]. Western blots show that NCOA4 expression is significantly increased at the peak and progressive stages of CH-EAE (Fig. 2Av and Additional file 2: Fig. S2Av; details of statistics for all data are provided in figure legends).

**Fig. 3:**
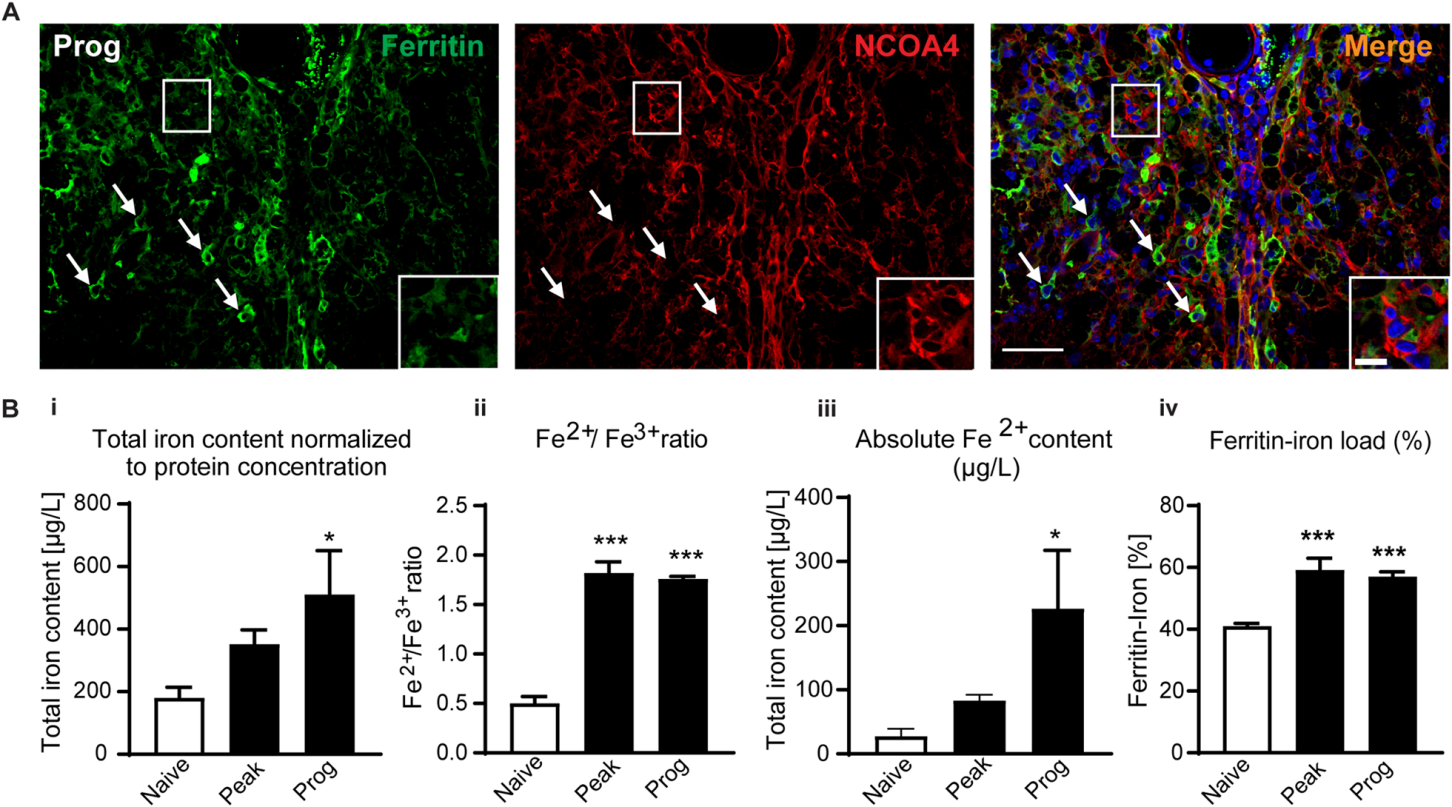
Expression of NCOA4 and ferritin, and Fe^2+^/Fe^3+^ iron levels in CH-EAE. **A** Double immunofluorescence labeling of rabbit anti-ferritin and mouse anti-NCOA4 shows that cells strongly expressing NCOA4 have reduced ferritin staining (suggesting ferritinophagy) (cells in rectangle, and in inset), while cells that express low levels of NCOA4 show high ferritin staining (arrows). **B** Data from CE-ICP-MS assay showing **Bi** total iron content in spinal cord normalized to protein concentration. Shows incremental increase in iron from naïve to peak and progressive stages of CH-EAE; naïve versus prog*: p* = *0.02; F*_*(2, 11)*_ = *4.5, p* = *0.037*. **Bii** the ratio of Fe^2+^/Fe^3+^ which indicate about 3.5-fold increase in redox active iron in the spinal cord at the peak and progressive stages compared to naïve (normal) spinal cord; naïve versus peak and prog: *p* < *0.001; F*_*(2, 12)*_ = *92.60, p* < *0.001*. **Biii** Absolute Fe^2+^ content in µg/L. Graph shows an incremental increase in Fe^2+^ content with disease progression; naïve versus prog*: p* = *0.05; F*_*(2, 12)*_ = *3.74, p* = *0.05*. **Biv** Graph showing the amount of iron loaded into ferritin. Data shows increase in the percentage of iron loading of ferritin in CH-EAE from ~ 40% in naïve control samples to ~ 60% at the peak and progressive stages: naïve versus peak and prog: *p < 0.001 and p = 0.001; F*_*(2, 12)*_* = 17.15, p < 0.001. n = 4–5* per group, one-way ANOVA with Bonferroni’s multiple comparison test. *p < 0.05, **p < 0.01 and ***p < 0.001. Scale bars: 40 µm; Inset:10 µm

We have previously reported that TfR1 is expressed in astrocytes and CD11b^+^ macrophages in EAE lesions, while DMT1 is expressed mainly in GFAP^+^ astrocytes [20]. Astrocytes, however, generally fail to accumulate iron in neurological disorders, suggesting that they have a good capacity to release or export iron likely via astrocytic end-feet around blood vessels [16] They also possess less of the ferritin light chain required for iron storage [16]. Ferritin, however, is expressed predominantly in CD11b^+^ macrophages in and near EAE lesions [20]. Iron accumulation also appears to be seen predominately in macrophages/microglia and oligodendrocytes [14, 20]. Therefore, cell counts for ferritin, HO-1, and NCOA4 were limited to CD11b+ macrophages and CC1+ oligodendrocytes. These counts were done at the disease stage when these molecules showed the highest level of expression by Western blots. Here we show that ferritin is expressed in 53.1 ± 4.0% of CD11b^+^ macrophages (Fig. 2B) and in 37.6 ± 3.9% of CC1^+^ oligodendrocytes in the progressive stage of EAE (Fig. 2C). On the other hand, in the progressive stage, 61.0 ± 2.5% of ferritin+ cells are CD11b + macrophages while only 24.0 ± 0.76% are CC1+ oligodendrocytes. We also show that HO-1 is expressed in 41.4 ± 2.68% and 54 ± 3.3% of CD11b^+^ macrophages at the peak and progressive stages of CH-EAE but in only 10.6 ± 0.7% of macrophages at the onset stage (Fig. 2D). Furthermore, NCOA4, which mobilizes iron stored in ferritin, is expressed in 49.6 ± 4.4% of CD11b^+^ macrophages (Fig. 2E) and in 42.8 ± 3.5% of CC1^+^ oligodendrocytes (Fig. 2F) at and near EAE lesions in the progressive stage of EAE. Additionally, 44.6 ± 1.5% of NCOA4+ cells are CD11b+ macrophages, while 31.7 ± 3.6% are CC1+ oligodendrocytes. These data and the ferritin cell counts mentioned above suggest strongly that macrophages are likely to be the main cell type contributing to redox active iron and ferroptosis in EAE. Moreover, cells that are strongly NCOA4^+^ show reduced or no ferritin staining (suggesting ferritinophagy), while cells that express low levels of NCOA4 show strong ferritin staining (Fig. 3A). Taken together, these results indicate that increased iron load (detected by ferritin labeling, a good surrogate marker for iron) occurs in macrophages and oligodendrocytes in CH-EAE, and that this ferritin-bound iron can be mobilized by NCOA4 to release redox active iron, setting up the conditions for ferroptosis. To assess if this occurs, we next assessed the levels of Fe^2+^ and Fe^3+^ iron in different stages of CH-EAE.

### Fe^2+^/Fe^3+^ redox speciation in CH-EAE

Estimating the amount of redox active ferrous iron in CH-EAE spinal cord will provide a good measure of the capacity for ferroptosis. To do this, we used a novel Capillary Electrophoresis coupled to Inductively coupled Plasma Mass Spectrometry (CE-ICP-MS) technique that can accurately and rapidly quantify total iron levels and Fe^2+^ and Fe^3+^ redox speciation in the spinal cord of normal and CH-EAE mice [44, 45]. Additionally, this analysis can also reveal how much Fe^3+^ iron is stored in ferritin. This analysis showed a gradual increase in total iron content in the spinal cord at the peak and progressive stages of CH-EAE compared to normal (naïve) mice, reaching significance at the later progressive stage (Fig. 3Bi). Importantly, CE-ICP-MS also revealed ~ 3.5-fold increase in the Fe^2+^/Fe^3+^ ratio at the peak and progressive stages of CH-EAE, as compared to the uninjured (naïve) spinal cord, indicative of increased amounts of redox active Fe^2+^ iron (Fig. 3Bii). There was no difference in this ratio between the peak and progressive stages (Fig. 3Bi) and correlates well with the increased expression levels of NCOA4 (Fig. 2Av). The absolute content of ferrous iron (in µg/L) also showed a gradual increase at the peak to the progressive stages, reaching ~ 8.0-fold increase at the progressive stage compared to naïve mice (Fig. 3Biii). These findings highlight that redox active iron increases with progression of CH-EAE that can promote ferroptosis.

Each ferritin molecule has the capacity to store up to 4500 atoms of iron [50]. We measured how much iron is stored in ferritin and estimated the amount of ferritin in the tissue by ELISA. This type of analysis showed that in normal (naïve) animals ~ 40% of the ferritin in the spinal cord is loaded with iron (Fig. 3Biv). This capacity increases to ~ 60% at the peak and progressive stages (Fig. 3Biv). These results show that additional iron is stored in ferritin in CH-EAE that can be released by ferritinophagy and fuel ferroptosis and oxidative damage.

### Changes in fatty acid metabolism markers of ferroptosis

**Fig. 4:**
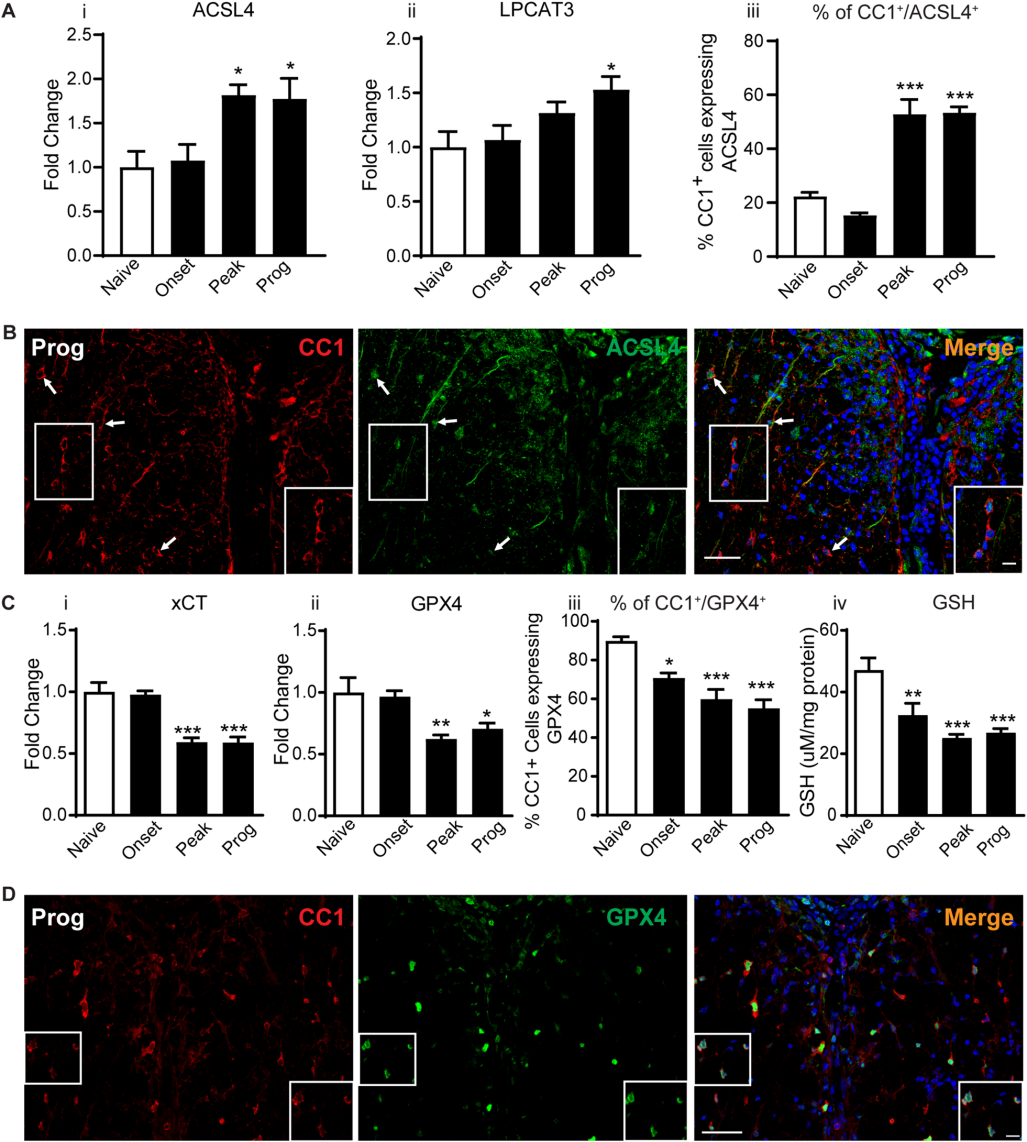
Changes in expression levels and cellular localization of ferroptosis markers related to lipid repair and GSH metabolism. **A** Western blot analysis of ACSL4 (**Ai**) and LPCAT3 (**Aii)** show increased expression in later stages of CH-EAE. (**Ai** ACSL4, naïve vs. peak and prog: *p* = 0.03 and 0.04 respectively; *F*_*(3, 16)*_* = 5.81, p = 0.007*; **Aii** LPCAT3, naïve vs. prog: *p = 0.04; F*_*(3, 16)*_* = 3.755, p = 0.03)*. **Aiii** The percentage of CC1^+^ oligodendrocytes expressing ACSL4 increases significantly from naïve to the peak *and progressive* stages of CH-EAE (naïve vs. peak and prog:* p* < *0.001; F*_*(3,16)*_ = *42.65, p* < *0.001*) **B** Double immunofluorescence labeling shows that ACSL4 is expressed in CC1^+^ oligodendrocytes. Expression in oligodendrocytes is increased in the peak and progressive stages of CH-EAE. **C** Western blot analysis of xCT (Ci) and GPX4 (**Cii**) show reduced expression in the peak and progressive stages of CH-EAE (***Ci:***  xCT; naïve vs. peak and prog: *p* = *0.009 and p* = *0.04 respectively; F*_*(3, 16)*_ = *6.952, p* = *0.003, Cii:*  GPX4; naïve vs. peak and prog: *p* < *0.001; F*_*(3, 16)*_ = *20.67, p* < *0.001*) Panel **Ciii** quantification showing a gradual reduction in the percentage of CC1^+^ oligodendrocytes expressing GPX4 (naïve vs. onset, peak and prog: *p* = *0.02 and p* < *0.001; F*_*(3, 12)*_ = *17.32, p* < *0.001*) Ai-iii and Ci-iii: *n* = 4–5 per group, one-way ANOVA with Tukey’s multiple comparison test. *p < 0.05, **p < 0.01 and ***p < *0.001*. Panel **Civ** shows reduction in GSH levels at all stages of CH-EAE. (Naïve vs. onset, peak and prog: *p* = *0.01 and p* < *0.001 respectively; F*_*(3, 20)*_ = *11.90, p* < *0.001, n* = *6 *per group, one-way ANOVA with Tukey’s multiple comparison test, **p < 0.01 and ***p < *0.001)*. **D** Double immunofluorescence co-localization of GPX4 in CC1^+^ oligodendrocytes. Scale bars: 40 µm; Inset: 12 µm

The repair (replacement) of oxidized fatty acids at the *sn*-2 position of membrane phospholipids can provide additional new targets for ferroptosis and promote more lipid peroxidation [16]. We therefore assessed the changes in expression at the protein level of two enzymes (ACSL4 and LPCAT3) involved in the incorporation of arachidonic acid into membrane phospholipids [51, 52]. Interestingly, these enzymes, which are known to promote ferroptosis [53, 54] are increased at the peak and progressive stages of CH-EAE (Fig. 4Ai, ii). ACSL4 is increased 1.8-fold at the peak and progressive stages (Fig. 4Ai and Additional file 2: Fig. S2Avi), while LPCAT3 is increased 1.5-fold at the progressive stage of CH-EAE (Fig. 4Aii and Additional file 2: Fig. S2Avii). These results support some earlier reports [31] and point to additional ways to induce ferroptosis in the later stages of CH-EAE. Double immunofluorescence labeling shows that ACSL4 is expressed in 15.3 ± 0.8%, 52.8 ± 5.4% and 53.4 + 2.1% of CC1^+^ oligodendrocytes at the onset, peak and progressive stages, respectively (Fig. 4Aiii). Little labeling of ACSL4 was detected in CD11b^+^ macrophages, except for staining of some cell processes (data not shown).

### Changes in the glutathione antioxidant pathway

For ferroptosis to occur, increase in redox active iron needs to be coupled with an insufficient antioxidant response in the glutathione pathway. This includes reduced expression of the antiporter xCT required to transport cystine into cells to produce glutathione, reduced GPX4, the enzyme that utilizes glutathione to scavenge lipid radicals [47]. Western blot analysis shows that the expression levels of xCT and GPX4 are reduced at the peak and progressive stages of CH-EAE (Fig. 4Ci–ii and Additional file 2: Fig. S2Aviii-ix). Double immunofluorescence labeling shows that there is a significant reduction in the number of CC1^+^ oligodendrocytes expressing GPX4 in the peak and progressive stages of CH-EAE; being reduced from 89.9 ± 2.0% in naïve mice, to 55.1 ± 4.4% in the progressive stage (Fig. 4Ciii). GPX4 was expressed in only 4.3 ± 0.56% CD11b + macrophages at the peak stage of CH-EAE. Finally, GSH levels measured using the Glutathione Assay kit from Cayman Chemicals showed reduced levels at all stages of the disease (Fig. 4Civ). There was about a 50% reduction in GSH at the peak and progressive stages of the disease compared to naïve levels (Fig. 4Civ).

### Lipid peroxidation in CH-EAE

**Fig. 5:**
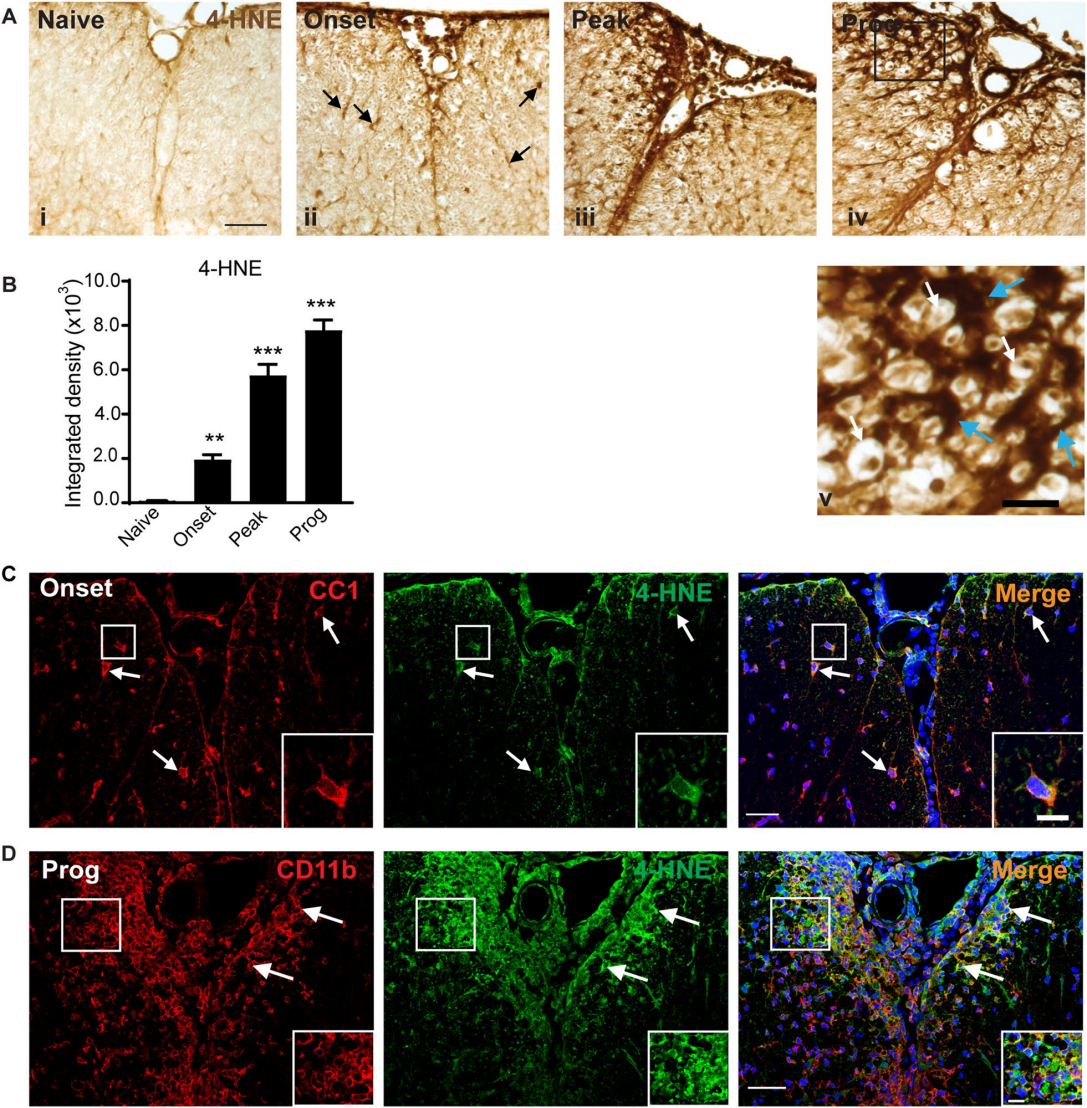
Lipid peroxidation in the spinal cord in CH-EAE. **A** Changes in 4-HNE labeling in the spinal cord in normal (naïve) mice and mice at different stages of CH-EAE detected by DAB-immunohistochemistry. Note the lack of 4-HNE labeling in naïve spinal cord (**Ai**), and gradually increasing labeling at the onset (**Aii**), peak (**Aiii**) and progressive (**Aiv**) stages of the disease. Panel **Av** shows higher magnification of the area outlined in the black rectangle in Aiv. In panel Av, note the dark 4-HNE labeling of glial processes (blue arrows) that surround myelinated axons (white arrows). Scale bar in Ai: 30 µm; Av: 10 µm. **B** Graph shows quantification by densitometry of the 4-HNE labeling. Note the progressive increase in 4-HNE with progression of the disease. Naïve versus onset, peak and prog: *p* = *0.008 and p* < *0.001* respectively;* F*_*(3, 16)*_ = *100.2, p* < *0.001, n* = *5 *per group, one-way ANOVA with Tukey’s multiple comparison test, **p < 0.01 and ***p < *0.001*. **C-D** Double immunofluorescence labeling shows co-localization of 4-HNE labeling to CC1^+^ oligodendrocytes at the onset stage (**C**) and CD11b^+^ macrophages at the progressive stage of CH-EAE (**D**). Double-labeled cells are outlined in the white rectangles and shown at higher magnification in bottom right of each panel. Scale bars: 40 µm; Inset: 12 µm

As lipid peroxidation is a key indicator of ferroptosis, we next assessed whether the increases seen in iron accumulation and reduction in GSH and related enzymes are associated with increased lipid peroxidation in CH-EAE. Lipid peroxidation of membrane phospholipids in plasma membranes, organelles, and myelin generate 4-hydroxy-2-nonenal (4-HNE), a highly toxic product. We assessed 4-HNE by quantitative immunohistochemistry using DAB as the chromogen. Little detectable staining is observed in normal, naïve spinal cord but 4-HNE immunoreactivity is detected in the spinal cord at the onset stage and increases further at the peak and progressive stages of CH-EAE (Fig. 5Ai–v). This staining appeared in discreet cells at the onset stage (arrows in Fig. 5Aii) that are likely to be oligodendrocytes, as significant immune infiltration is not observed at this early stage. Staining is markedly increased at the peak and even more in the progressive stages of CH-EAE, when it is seen in what appear to be the resident glia and some infiltrating cells, including staining surrounding axons that suggest lipid peroxidation in oligodendrocytes and myelin sheaths at the lesion site (Fig. 5Aiv–v). Densitometric quantification of the 4-HNE staining shows ~ twofold increase at the onset of disease, followed by ~ sixfold and sevenfold increase at the peak and progressive stages, respectively (Fig. 5B). Interestingly, at the onset stage when there are few infiltrating immune cells, 14.2 ± 2.3% of CD11b+ macrophages and 40.2 ± 3.9% of CC1^+^ oligodendrocytes were 4HNE^+^, while at the progressive stage 55.9 ± 1.8% of CD11b^+^ macrophages and 29.8 ± 3.2% of CC1+ oligodendrocytes were 4HNE^+^ at sites of EAE lesions (Fig. 5D). These data match with the evidence that macrophages are the main cell type that express ferritin and NCOA4.

### Ferroptosis inhibitor treatment improves clinical and histological outcomes

**Fig. 6:**
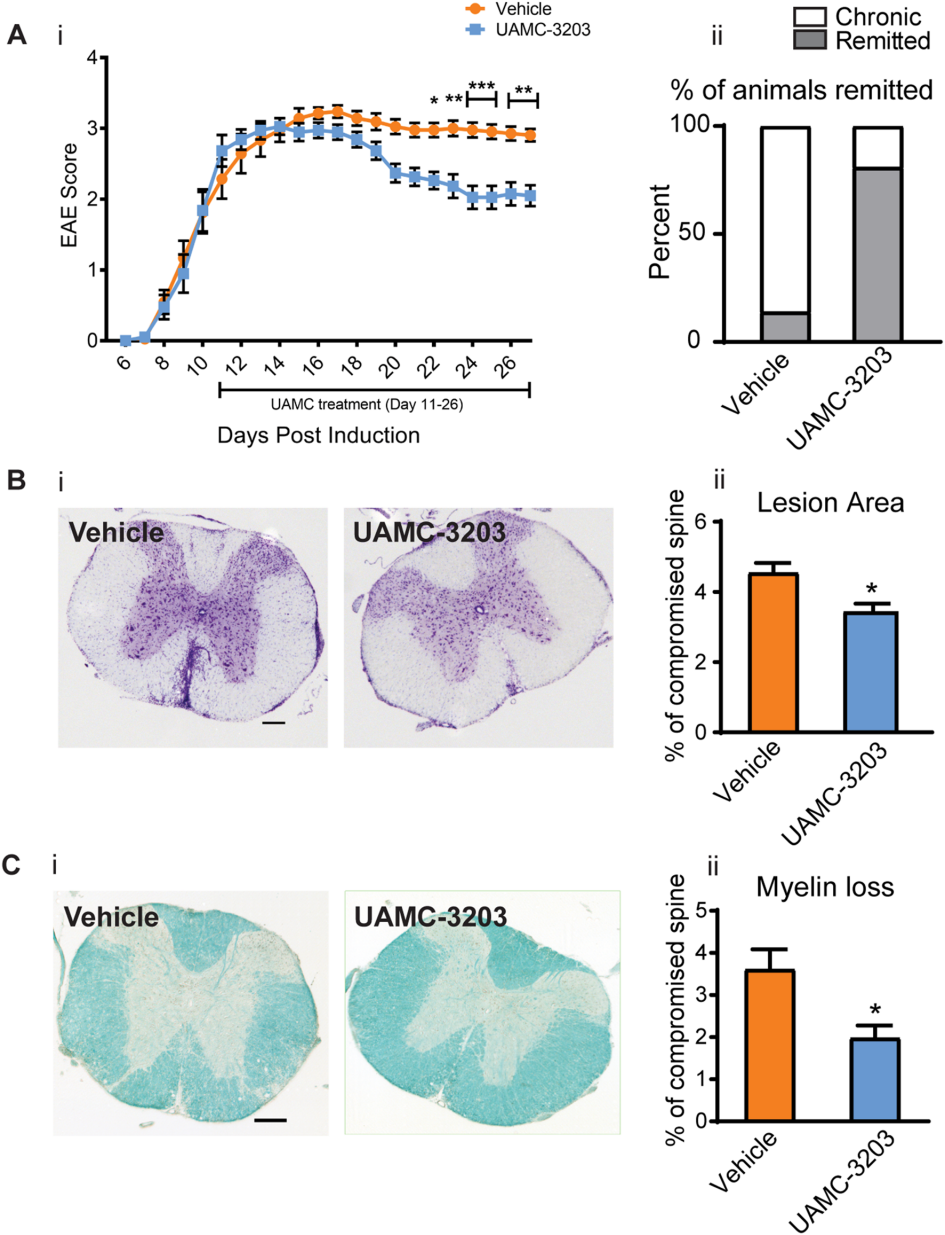
Ferroptosis inhibitor treatment improves clinical and histological outcomes in CH-EAE. (**Ai**) Graph showing population scores of EAE paralysis of pooled data from 2 experiments. Note the UAMC-3203 treated mice show significant improvement from day 22. Vehicle treated vs. UAMC treated at day 22: *p* = *0.03; day23: p* = *0.007*; day 24 & 25*: p* < *0.001*; day 26 & 27*: p* = *0.003; F*_*(1,838)*_ = *48.34, p* < *0.001, n* = *19–21 *per group, two-way ANOVA with Bonferroni’s multiple comparison test. **Aii** Shows the number of animals that remitted to a score of 2 or below. This analysis reveals that only 14% of the vehicle treated mice remitted while about 81% of the UAMC-3203 treated group remitted. **Bi** Cresyl violet staining of cross-sections of the thoracic spinal cord from vehicle and UAMC-3203 treated mice at 27 days after immunization. Note the cellular infiltrates in the EAE lesions in the vehicle treated group . **Bii** Quantification of the size of the lesions shows significant reduction in the UAMC-3203 treated group. **Ci** LFB staining of thoracic spinal cord cross-sections from vehicle and UAMC-3203 treated group. Note the demyelinated areas in the vehicle treated group . **Cii** Quantification of LFB staining shows a significant reduction in myelin loss in the UAMC-3203 treated group. Bii and Cii: Vehicle treated versus UAMC treated: *p* = 0.01 (Bii) and p = 0.02 (Cii). n = *4–5 *per group, Mann–Whitney test, *p < *0.05*. Scale bars: 35 µm

The data presented above provide strong evidence that ferroptosis is likely to occur in CH-EAE. To obtain more direct evidence, we treated CH-EAE mice daily with a ferroptosis inhibitor (UAMC-3203) for 15 days intraperitoneally. Note that treatment was started only after mice reached the peak of clinical paralysis (i.e., score of ≥ 3). UAMC-3203 is a lipid radical scavenging agent that was designed based on the scaffold of the first generation of ferroptosis inhibitor (Fer-1) and shown to have better pharmacokinetics [39]. CH-EAE mice treated with UAMC-3203 have population scores that show significant reduction in clinical paralysis starting from day 11 after start of treatment (i.e., 22 days post-immunization) (Fig. 6Ai). We have previously proposed that CH-EAE mice that show a drop in score to grade 2 or less are considered to have remitted [36, 37]. When scores of individual mice are assessed on this criterion, 81% of CH-EAE mice treated with UAMC-3203 show remission, while only 14% of vehicle treated mice remit (Fig. 6Aii).

**Fig. 7:**
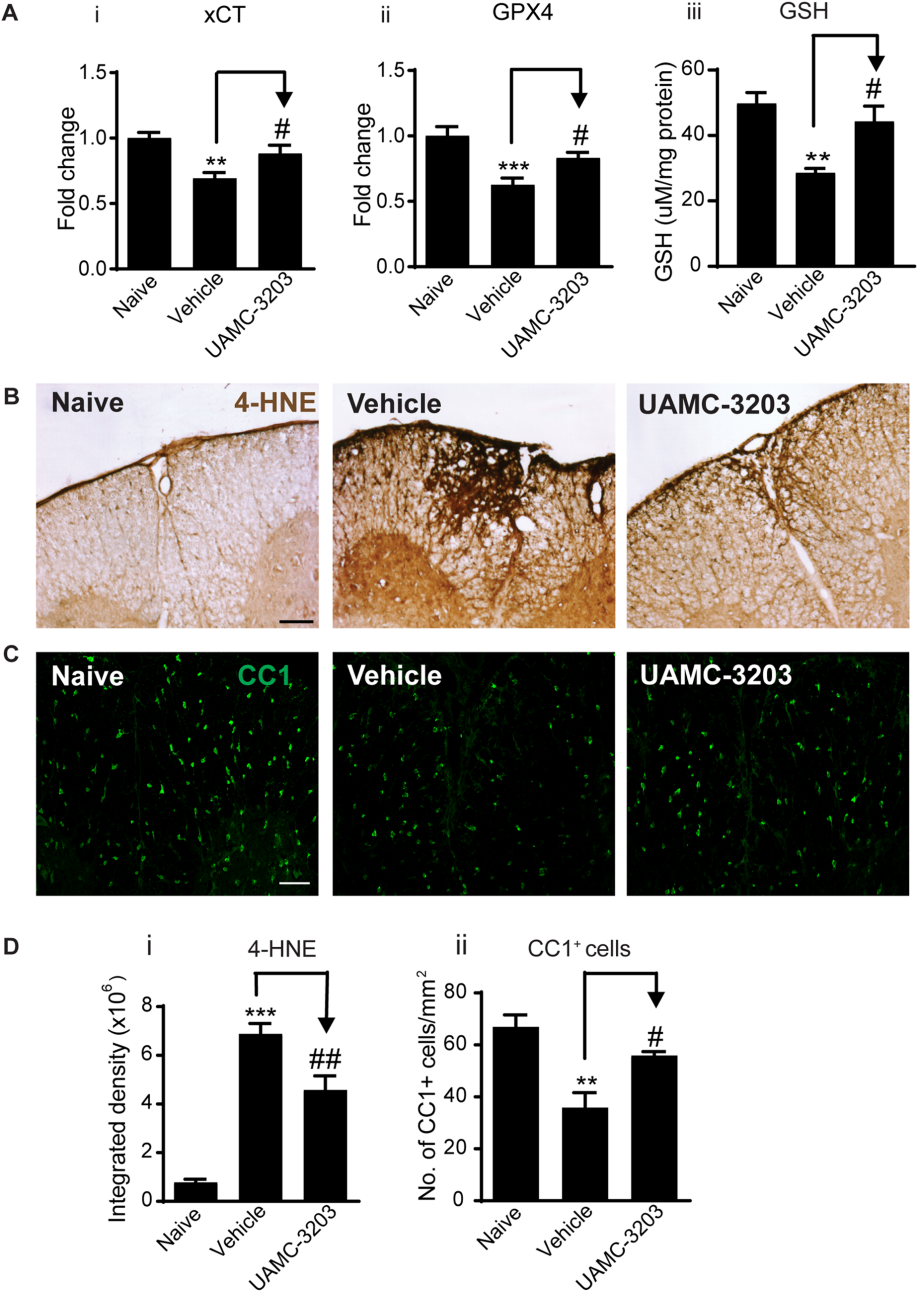
UAMC-3203 treatment induces improvement in other outcome measures. **A** Western blot data show increased expression of xCT (**Ai**) and GPX4 (**Aii**) in UAMC-3203 treated as compared to vehicle treated CH-EAE mice. (**Aiii**) GSH levels measured by a colorimetric method also increase in the UAMC-3203 treated group as compared to vehicle treated CH-EAE mice. **B** DAB-immunohistochemical staining of 4-HNE in naïve, vehicle treated, and UAMC-3203 treated CH-EAE mice. Note the reduction in 4-HNE labeling in the UAMC-3203 treated mice. Quantification of this labeling shows significant reduction in 4-HNE in the inhibitor treated group as compared to vehicle treated CH-EAE mice (**Di**). **C** Immunofluorescence labeling of CC1^+^ oligodendrocytes in naïve, vehicle treated, and UAMC-3203 treated CH-EAE mice. Quantification of CC1^+^ cells indicate a significant increase in the UAMC-3203 treated mice compared to vehicle treated CH-EAE mice (**Dii**). Scale bar in B: 40 µm; C: 60 µm. (***Ai*** xCT: Naïve versus Vehicle treated: *p* = *0.002;* Vehicle treated vs. UAMC-3203 treated: *p* = *0.048; F*_*(2, 15)*_ = *9.129, p* = *0.0022. Aii.* GPX4: Naïve versus Vehicle treated: *p* < *0.001;* Vehicle treated vs. UAMC-3203 treated: *p* = *0.048; F*_*(2, 15)*_ = *11.30, p* = *0.001. *For Ai and Aii:* n* = *6 *per group, one-way ANOVA with Tukey’s multiple comparison test. **Aiii** GSH level: Naïve versus Vehicle treated: *p* = *0.002;* Vehicle treated versus UAMC-3203 treated: *p* = *0.02; F*_*(2, 12)*_ = *10.33, p* = *0.002; n* = *5 *per group, one-way ANOVA with Tukey’s multiple comparison test. **Di**. Naïve versus Vehicle and UAMC-3203 treated: *p* < *0.001; F*_*(2, 12)*_ = *254.8, p* < *0.001; n* = *5 *per group, one-way ANOVA with Tukey’s multiple comparison test, **Dii**. Naïve versus Vehicle treated: *p* = *0.002;* Vehicle treated vs. UAMC-3203 treated: *p* = *0.02; F*_*(2, 9)*_ = *13.13, p* = *0.002, n* = *4 *per group, one-way ANOVA with Tukey’s multiple comparison test,*,^*#*^*p* < 0.05,**^,##^*p* < 0.01 and ***p < *0.001*

Furthermore, histological analysis showed significant reduction in lesion size as detected by Nissl stain (Fig. 6Bi, ii), and reduced myelin loss as detected by LFB staining (Fig. 6Ci, ii). Importantly, UAMC-3203 treatment significantly increased the protein levels of xCT, GPX4 (Fig. 7Ai–ii and Additional file 2: Fig. S2Bi-ii) and GSH (Fig. 7Aiii) and resulted in a significant reduction in lipid peroxidation (4-HNE staining) at and near EAE lesions (Fig. 7B and Di). Additionally, UAMC-3203 treatment also significantly increased the number of CC1^+^ oligodendrocytes in similar regions of the spinal cord (Fig. 7C and Dii).

### Expression of key ferroptosis markers in SPMS

**Fig. 8:**
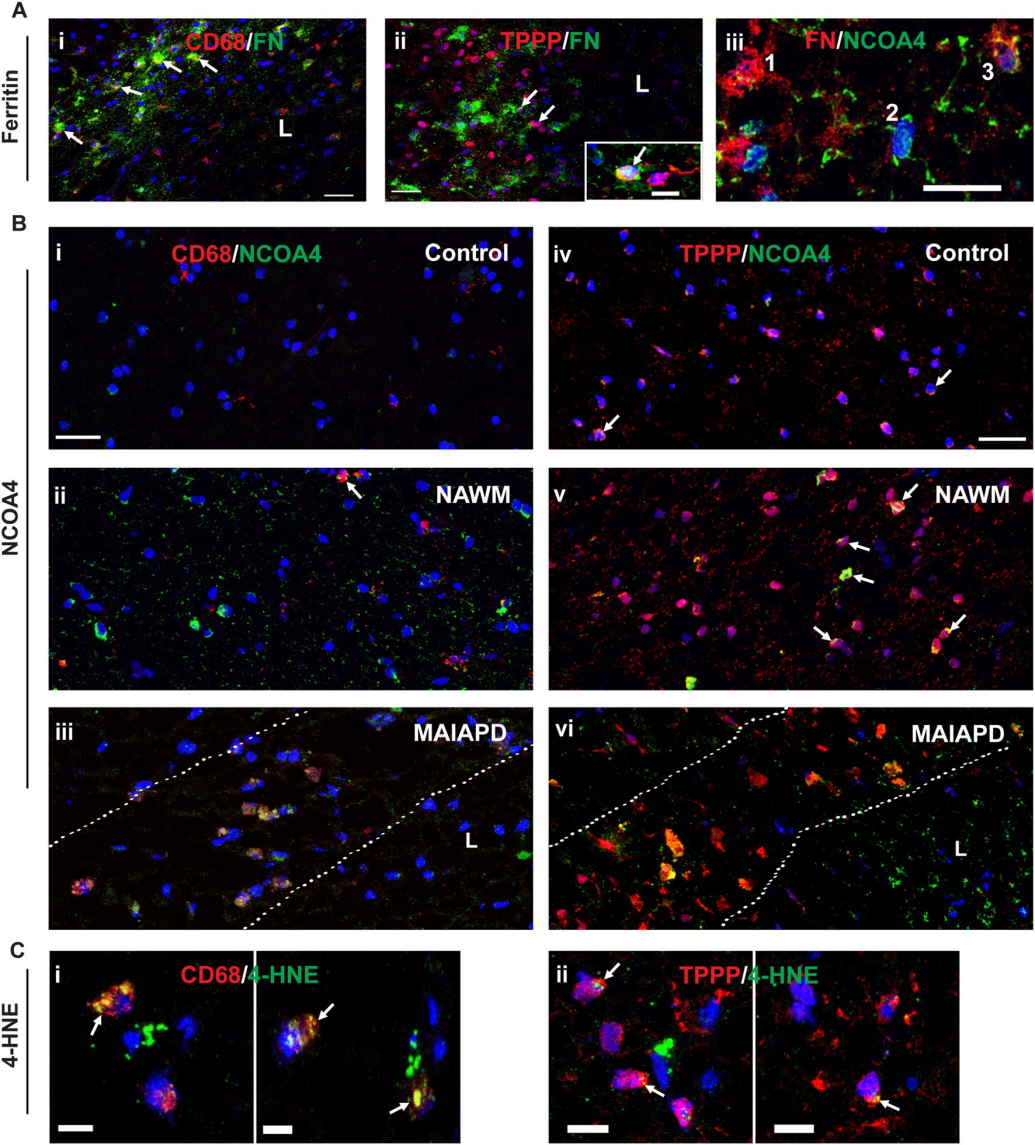
Expression of key ferroptosis markers in SPMS. **A** Double immunofluorescence labeling of ferritin (FN) in macrophages (CD68/FN) (**Ai**); and oligodendrocytes (TPPP/FN) (**Aii**). All immunofluorescence sections are stained with nuclear DAPI stain; L = lesion. Note the clear expression of ferritin in macrophages lined up along the lesion rim (arrows) (**Ai**). In contrast, fewer oligodendrocytes showed double labeling of TPPP and FN (**Aii**). FN labeling surrounds some TPPP^+^ cell bodies and may represent FN in cell processes (arrows). Inset shows double labeling of TPPP and FN (arrow) in another section. **Aiii** Shows double labeling of ferritin and NCOA4 (FN/NCOA4). Note that cells showing strong FN labeling express little or no NCOA4 (1), and cells that show no ferritin strongly express NCOA4 (2), while cells that express some NCOA4 express lower levels of FN (3). **B** Panels showing double labeling of CD68 and NCOA4 (i-iii) and TPPP and NCOA4 (iv-vi) in Controls (i and iv), NAWM (ii and v) and MAIAPD (iii and vi). All panels stained with DAPI. **Bi–Biii** In CD68+ and NCOA4 staining, note the absence of CD68^+^ macrophages and lack of NCOA4 in Control tissue. **Bii** In NAWM only about 10% of the CD68^+^ macrophages express NCOA4 (arrow). **Biii** In contrast, CD68^+^ macrophages lining the rim of MAIAPD lesions were double labeled with NCOA4. The hatched lines demarcate the lesion rim. **Biv–Bvi** In TPPP^+^ and NCOA4 staining, in control samples, a small number TPPP^+^ oligodendrocytes express NCOA4 (arrows). **Bv** The numbers and staining intensity of NCOA4 increased in NAWM (arrows). **Bvi** In contrast, NCOA4 expression is markedly increased in TPPP^+^ oligodendrocytes along the lesion rim located between the hatched lines. **C** Panels showing CD68^+^ macrophages (**Ci**) and TPPP^+^ oligodendrocytes (**Cii**) double labeled with 4-HNE (arrows). Note that the 4-HNE staining is stronger in macrophages as compared to the sparce labeling in oligodendrocytes. Scale bar in A and B: 30 µm; C and Inset: 10 µm

We next carried out experiments to extend these findings in mice to SPMS in humans. Recent studies have reported that chronic active lesions in SPMS are surrounded by a rim of iron containing macrophages, called iron rim lesions (IRLs). These lesions expand over time as compared with non-IRLs which shrink [7–11]. Therefore IRLs in SPMS are significantly more destructive than non-IRLs [7, 15], and occur in the absence of signs of peripherally mediated inflammation [7, 8, 55]. Iron in these macrophages is generally stored safely in ferritin. Therefore, for this iron to become destructive it needs to be released from ferritin. One way for this to occur is by increased expression of NCOA4 which shuttles ferritin to autophagosomes for degradation and release of redox active iron [16]. In addition to macrophages, iron can also be found in oligodendrocytes, which require it for myelination [56]. We therefore first examined the expression of ferritin in SPMS lesions as well as NAWM and control subjects. We identified mixed active/inactive actively demyelinating (MAIAD) and mixed active/inactive and post-demyelinating (MAIAPD) lesions in SPMS tissue samples stained with Luxol fast blue/Hematoxylin and Eosin as well as Oil Red O (LHE-ORO) (Additional file 3: Fig. S3A, B) according to the classification by Kuhlmann et al. [10]. The two types of lesions were distinguished based on the LHE and ORO histological staining, as the MAIAD lesions show ORO staining, while the MAIAPD lesions do not show ORO staining. Both lesions show myelin loss (detected by loss of Luxol fast blue staining). Histology of the LHE and ORO staining is shown in Additional file 3: Fig. S3A and B. A total of 11 lesions from 5 SPMS cases and CNS tissue from 4 controls were assessed (see Table 1 for more details). The 11 lesions include 4 MAIAD and 7 MAIAPD lesions. Both types of lesions show macrophages along the rim of the lesion [10]. Ferritin^+^ cells were detected along the rim of these SPMS lesions and found to be localized mainly to CD68^+^ macrophages (Fig. 8Ai) and in some TPPP^+^ oligodendrocytes (Fig. 8Aii). Ferritin is a good surrogate marker for iron as its expression is regulated at the mRNA level via the IRP-IRE system by which elevated amounts of intracellular iron increases ferritin mRNA expression and iron storage [16]. Furthermore, iron positive cells are detected along the rim of the lesions by Turnbull’s iron histochemistry (Additional file 3: Fig. S3C). These findings suggest that ferritin-bound iron is likely to be located mainly in CD68^+^ macrophages. Along the rim of the lesions, CD68^+^ macrophage numbers were increased about fivefold as compared to control samples and NAWM.

As our animal work indicates that CD11b+ and CC1+ cells are the major cell types that express ferritin and NCOA4, we focused our attention on these two cell types in the human samples. Therefore, we next assessed the expression of NCOA4 in CD68^+^ macrophages. Double immunofluorescence labeling revealed that in control samples none of the few CD68^+^ cells showed NCOA4 labeling (Fig. 8Bi). In NAWM, however, a small number of CD68^+^ macrophages (10.8 ± 0.48%) were NCOA4^+^ (Fig. 8Bii). In contrast, there is a significant increase of NCOA4 expression in CD68^+^ cells in MAIAD (30.8 ± 1.4%) and MAIAPD (38.5 ± 5.5%) lesions in SPMS (Fig. 8Biii and Additional file 4: Fig. S4Ai), with no significant differences between these groups (Additional file 4: Fig. S4Ai). The pooled average of these 2 SPMS groups is 35.4 ± 3.4% (Additional file 4: Fig. S4Aii). The NCOA4^+^/CD68^+^ cells are located along the rim of the lesions (Fig. 8Biii). Interestingly, two of the lesions in the latter group (MAIAPD) had 57.8% and 50% CD68^+^/NCOA4^+^ double labeled cells.

With regards to oligodendrocytes, 16.3 ± 0.63% of TPPP^+^ oligodendrocytes expressed NCOA4 in control samples, while in NAWM, 25.3 ± 2.6% of TPPP^+^ cells expressed NCOA4. Although this difference in number of NCOA4^+^ oligodendrocytes was not statistically significant between the two groups (Additional file 4: Fig. S4Bi), the intensity of the NCOA4 labeling appeared to be higher in NAWM (Fig. 8Biv–v). In contrast, there is a significant increase in the number of TPPP^+^ cells expressing NCOA4 along the rim of MAIAD (38.0 ± 3.7%) and MAIAPD (36.7 ± 1.3%) lesions in SPMS (Fig. 8Bvi and Additional file 4: Fig. S4Bi). The difference between these lesions was not statistically significant and the pooled mean value being 37.2 ± 1.5% (Additional file 4: Fig. S4Bii).

Interestingly, cells that strongly express NCOA4 show little or no ferritin labeling (Fig. 8Aiii), with a threefold increase in such cells in SPMS lesion as compared to controls (11.4 ± 4.3% [control] vs. 36.1 ± 2.6% [SPMS]. In contrast, cells stained strongly for ferritin had little or no NCOA4 labeling (Fig. 8Aiii). These results suggest that the expression of NCOA4 appears to be associated with reduced ferritin (ferritinophagy).

The release of redox active iron from ferritin can be expected to induce oxidative damage that can be detected as increased levels of 4-HNE. Double immunofluorescence labeling for 4-HNE/CD68 showed a sixfold increase in 4-HNE^+^ macrophages in SPMS lesions (8.1 ± 1.4 [Controls] vs. 49.4 ± 2.2 [SPMS]; latter value is the pooled average of MAIAD [45.5 ± 2.4] and MAIAPD [52.2 ± 2.9]) (Additional file 4: Fig. S4Ci and ii). The number in NAWM (13.1 ± 1.3) was not significantly different from Controls (Additional file 4: Fig. S4Ci and ii). In contrast to macrophages, far fewer TPPP^+^ oligodendrocytes showed 4-HNE labeling. Double labeling of 4-HNE/TPPP was found in only 1.15 ± 0.15% in Control samples and 2.1 ± 0.3% of NAWM. There was, however, a significant increase in SPMS lesion of 16.0 ± 2.2 (pooled average of MAIAD (14.7 ± 3.8) and MAIAPD (16.8 ± 2.9) (Additional file 4: Fig. S4Di and ii). The 4-HNE immunofluorescence labeling was stronger in macrophages (Fig. 8Ci) as compared to sparce labeling in oligodendrocytes (Fig. 8Cii).

These findings suggest that macrophages and possibly also oligodendrocytes along the rim of mixed active/inactive actively demyelinating (MAIAD) and post-demyelinating (MAIAPD) lesions may contribute to the liberation of redox active iron from ferritin and thus also contribute to lesion expansion in SPMS.

## Discussion

The two main requirements for ferroptosis include: (1) mechanisms that increase intracellular iron and mobilize redox active iron from ferritin; and (2) mechanisms that lead to an insufficient glutathione-mediated antioxidant defense required to prevent lipid peroxidation [23]. In this study we provide several lines of evidence of dysregulation in the expression of molecules involved in these two key pathways in CH-EAE. Furthermore, a small molecule ferroptosis inhibitor can significantly reduce the clinical and histological severity of CH-EAE when administered after the mice reach the peak of clinical EAE paralysis. In addition, histological analysis of CNS lesions in SPMS autopsy material revealed: (1) increased ferritin labeling (a surrogate marker of iron) in macrophages and oligodendrocytes along the rim of SPMS lesions (MAIAD and MAIAPD); (2) increased expression of NCOA4 (required for ferritinophagy and release of redox active iron), and (3) increased lipid peroxidation, in these cells, that suggest increased likelihood for ferroptosis that can contribute to progressive pathology in SPMS.

Increase in intracellular iron at sites of EAE and MS lesions can occur via increased iron uptake. Our Western blot analysis showed increased expression of iron uptake proteins, TfR1 (onset stage) and DMT1 (peak stage) in CH-EAE. Our earlier studies showed that TfR1 is expressed mainly in macrophage/microglia in EAE lesions [20]. TfR1 is also known to be expressed in endothelial cells, oligodendrocyte precursor cells (OPCs) and newly formed oligodendrocytes [57], which have a high requirement for iron for myelination [58]. In MS lesions, TfR1 expression is increased in peri-plaque white matter [14]. Thus, macrophages and cells of the oligodendrocyte lineage can uptake transferrin-bound iron via receptor-mediated endocytosis during the early onset stage of CH-EAE and in MS. We have previously shown that DMT1, another iron importer, is expressed mainly in astrocytes but also likely in other cells in EAE [20] and after CNS injury [59]. Uptake into astrocytes may be a way to clear some of the excess iron from the CNS via astrocytic end-feet, as astrocytes possess iron uptake and release mechanisms [47]. However, there is evidence of iron accumulation in some astrocytes in MS lesions [8]. DMT1 expression is also increased in glial cells in peri-plaque white matter around MS lesions [14]. Increased expression of these influx transporters can thus contribute to the increased iron load in glial cells in MS and CH-EAE.

Much of the iron in EAE and MS lesions is stored in macrophages/microglia which acquire iron from phagocytosed materials, such as oligodendrocytes and myelin. Expression of HO-1, which extracts Fe^2+^ iron from heme, reaches highest levels at the peak and progressive stages of CH-EAE, particularly in CD11b^+^ macrophages/microglia in and around the lesion when phagocytosis is likely at its peak. HO-1 has also been reported to be upregulated in macrophages in EAE [60]. Furthermore, HO-1 is strongly expressed in phagocytic foamy macrophages in active MS lesions [61] and HO-1 mRNA is highly enriched in MS samples [62]. Therefore, HO-1, which is crucial for the acquisition of iron by macrophages in MS and CH-EAE lesions, is upregulated in the later stages of the disease. A recent single-nucleus transcriptome analysis of chronic MS lesions identified two subsets of microglia—one associated with phagocytosis, enriched in foam-cell differentiation and lipid storage genes, and a second subset associated with iron metabolism, enriched among others in genes encoding heavy and light chain ferritin, and complement component C1 complex (*C1QA* and *C1QB*) [12]. The ferritin rich macrophage/microglia (ferritin^+^ cells) in SPMS that we describe here, are likely to belong to this second subset.

Normally, iron entering cells is utilized in mitochondria for biogenesis of iron-sulfur clusters and heme [63]. Excess iron in the cytosol is safely stored in ferritin in a non-redox active form [64]. The expression of ferritin is tightly regulated at the mRNA level by cellular iron levels via the iron response protein-iron response element (IRP-IRE) system [65]. Our Western blots show incremental increase in ferritin that reaches maximum levels at the progressive stage in CH-EAE. Ferritin has a high iron storage capacity and composed of 24 heavy and light chains that form a hollow sphere which holds up to 4,500 atoms of iron [66]. Interestingly, the novel CE-ICP-MS technique we used in our study revealed that the percentage of iron stored in ferritin is significantly increased from ~ 40% in normal mice to ~ 60% in CH-EAE mice, indicating increased iron burden in CH-EAE. Ferritin is expressed in macrophages in EAE [20] and MS [14]. We show here that it is expressed in about 53% of macrophages and 37% of CC1^+^ oligodendrocytes in and near CH-EAE lesions. Iron stored in ferritin is harmless, however, its detrimental effects arise when redox active iron is mobilized out of ferritin via ferritinophagy.

A key requirement for ferritinophagy is NCOA4 which binds to ferritin and shuttles it to autophagosomes for degradation [49]. Normally, this mechanism makes iron available for normal physiological needs [49]. However, high levels of expression of NCOA4 are detected in pathological conditions [47] which would induce release of excess amounts of redox active iron that can be cytotoxic. We recently showed a rapid and transient increase in expression of NCOA4 in oligodendrocytes, in the cuprizone-induced demyelination model [32]. This is associated with rapid reduction in ferritin (ferritinophagy) and increase in lipid peroxidation (4-HNE) and to ferroptosis-mediated loss of oligodendrocytes, which could be prevented with a ferroptosis inhibitor (Fer-1) [32]. Here we provide evidence that expression of NCOA4 increases significantly at the peak and progressive stages of CH-EAE. We also show that NCOA4 is expressed in about 49% of macrophages and 42% of oligodendrocytes in CH-EAE lesions, suggesting that redox active iron is likely to be mobilized from ferritin in these cell types. These findings could be further strengthened by studies on NCOA4 null mice.

Interestingly, our CE-ICP-MS analysis shows a 3.5-fold increase in the ratio of redox active Fe^2+^/Fe^3+^ iron at the peak and progressive stages of CH-EAE as compared to the normal spinal cord, and to an increase in the absolute amount of redox active Fe^2+^ iron. Such an increase in redox active iron can contribute to ferroptosis in CH-EAE. Furthermore, we also detect NCOA4 expression is increased in macrophage/microglia and oligodendrocytes along the rim of MAIAD and MAIAPD lesions in SPMS. Therefore, a key requirement for mobilizing redox active iron for ferroptosis is present in CH-EAE and SPMS. A recent study screening ferroptosis related genes in peripheral blood of RRMS cases reported down-regulation of NCOA4 [67]. This may indicate protection and promotion of remission in RRMS by preventing release of redox active iron from ferritin stores.

Ferroptosis also requires an insufficient glutathione response that normally protects against lipid peroxidation. Two major elements of this pathway are the enzyme xCT, which transports cystine into cells to produce GSH, and the enzyme GPX4, which utilizes GSH to prevent lipid peroxidation [47, 68]. An earlier study reported reduction of xCT and GPX4 at the peak of EAE [69]. Our more detailed Western blot analysis of three stages of CH-EAE show that expression of xCT and GPX4 are significant reduced at the peak and progressive stages but not at the onset stage. Expression of GPX4 in CC1^+^ oligodendrocytes decrease from 90% of cells in normal mice to 55% in the progressive stage. Furthermore, only 4.3% of CD11b^+^ macrophage/microglia express GPX4 at the peak stage in CH-EAE. These findings confirm that the two requirements for ferroptosis are met at the peak and progressive stages of CH-EAE. Importantly, glutathione peroxidase is marked reduced in the CSF of MS cases compared to controls [70]. However, others have reported increase in glutathione peroxidase in active demyelinating MS plaques [61], and increase in antioxidant enzymes (e.g., SOD1, HO-1, GPX4) in CD68^+^ microglia/macrophages in EAE and MS [60], which may reflect a protective response to lessen oxidative damage in certain lesions.

Importantly, there are reports of reduction in GSH in EAE [69, 71, 72]. Our results show about a 55% reduction in GSH at the peak and progressive stages of CH-EAE. Furthermore, there are also reports of significant reduction in GSH in the CSF of MS patients compared to normal subjects [73]. Additionally, a magnetic resonance spectroscopy study reported markedly lower levels of GSH in the frontotemporal cortex in SPMS and PPMS but no change in RRMS compared to controls [74]. Together, these findings provide strong evidence of insufficiency in the glutathione pathway in CH-EAE and progressive forms of MS.

The combined effect of increased redox active iron and reduced glutathione-mediated antioxidant defense will lead to an increase in lipid peroxidation. We see 4-HNE, a marker of lipid peroxidation, is increased between six and sevenfold in the late stages of CH-EAE and is localized mainly to macrophages and some oligodendrocytes along the rim of SPMS lesions (MAIAD and MAIAPD), indicating that they may be the main sites of ferroptosis. Increase in MDA and 4-HNE at the peak stage of EAE was also detected by spectrophotometry [69]. 4-HNE is also detected in MS in phagocytic macrophages and reactive astrocytes near active demyelinating lesions [61] as well as in oligodendrocytes and neurons [75, 76]. Interestingly, we also found that 4-HNE expression is increased in macrophage/microglia and some oligodendrocytes in both MAIAD and MAIAPD lesions in SPMS. Such oxidative damage has been proposed to contribute to the degenerative changes in progressive MS [77].

Ferroptosis can also be worsened by increased expression of enzymes that repair oxidized membrane phospholipids, thus providing new targets for ferroptosis. The enzymes ACSL4 and LPCAT3 play such roles by removing oxidized fatty acids and replacing them in cell membranes. We show that both enzymes are increased at the peak and progressive stages of CH-EAE. Increased expression of these enzymes can thus contribute to continued oxidation of newly regenerated membrane phospholipids and thus foster on-going ferroptosis and tissue damage in CH-EAE. ACSL4 was shown recently to induce degeneration in chronic EAE [31].

Ferroptosis can be selectively blocked by radical trapping antioxidants that scavenge lipid radicals and stop the propagation of lipid peroxidation [78, 79]. Ferrostatin-1 and liproxstatin-1 were the first generation ferroptosis inhibitors to be developed and have been tested in various animal models of neurological disease [16, 23]. We have shown that Fer-1 is effective in preventing oligodendrocyte death and demyelination in cuprizone-induced demyelination in the CNS in mice [32]. An important aspect of the current study is the use of a next generation ferroptosis inhibitor (UAMC-3203). UAMC-3203 is a small molecular weight (471 Da) lipophilic analogue of ferrostatin-1 with improved solubility and PK properties [39] and therefore expected to cross the blood–brain barrier (BBB). Reduced BBB permeability, however, was recently mentioned in a publication but no data provided [80] and requires further study. It is likely that permeability is increased at sites of CNS pathology, allowing for better entry at these sites. UAMC-3203 does not appear to bind to blood cells [80], suggesting that its effects in this model may not be mediated mainly by its actions on peripheral immune cell. Further work is needed to clarify these issues. We find that UAMC-3203 significantly reduces the population score of clinical paralyses in CH-EAE. Furthermore, 81% of mice treated with UAMC-3203 remitted to a score of 2 or less, compared to only 14% in the control group. Note also that treatment was started after the mice reached the peak of EAE paralysis (score of 3 or higher). This treatment also increased GSH levels and related enzymes, reduced lipid peroxidation, histological measures of tissue damage, and oligodendrocyte loss.

Our blocking experiments with UAMC-3203 in mice show that ferroptosis is likely to contribute to the degeneration and loss of function in CH-EAE. The findings in MS, along with the strong evidence from our pre-clinical work suggest the need to consider testing ferroptosis inhibitors for SPMS, for which there are limited treatment options.

## Supplementary Information

**Additional file 1: Figure 1**. EAE population scores.

**Additional file 2: Figure 2**. Western blots.

**Additional file 3: Figure 3**. LHE-ORO staining and iron histochemistry of human MS tissue sections.

**Additional file 4: Figure 4**. Quantification of double immunofluorescence labelled cells of Human tissue sections.

**Additional file 5**: Methods.
