# Supplementary figures and images for: Ferroptosis induces detrimental effects in chronic EAE and its implications for progressive MS
Source: Acta Neuropathol Commun. 2023 Jul 25;11:121. doi: 10.1186/s40478-023-01617-7 (PMC10369714; doi:10.1186/s40478-023-01617-7)

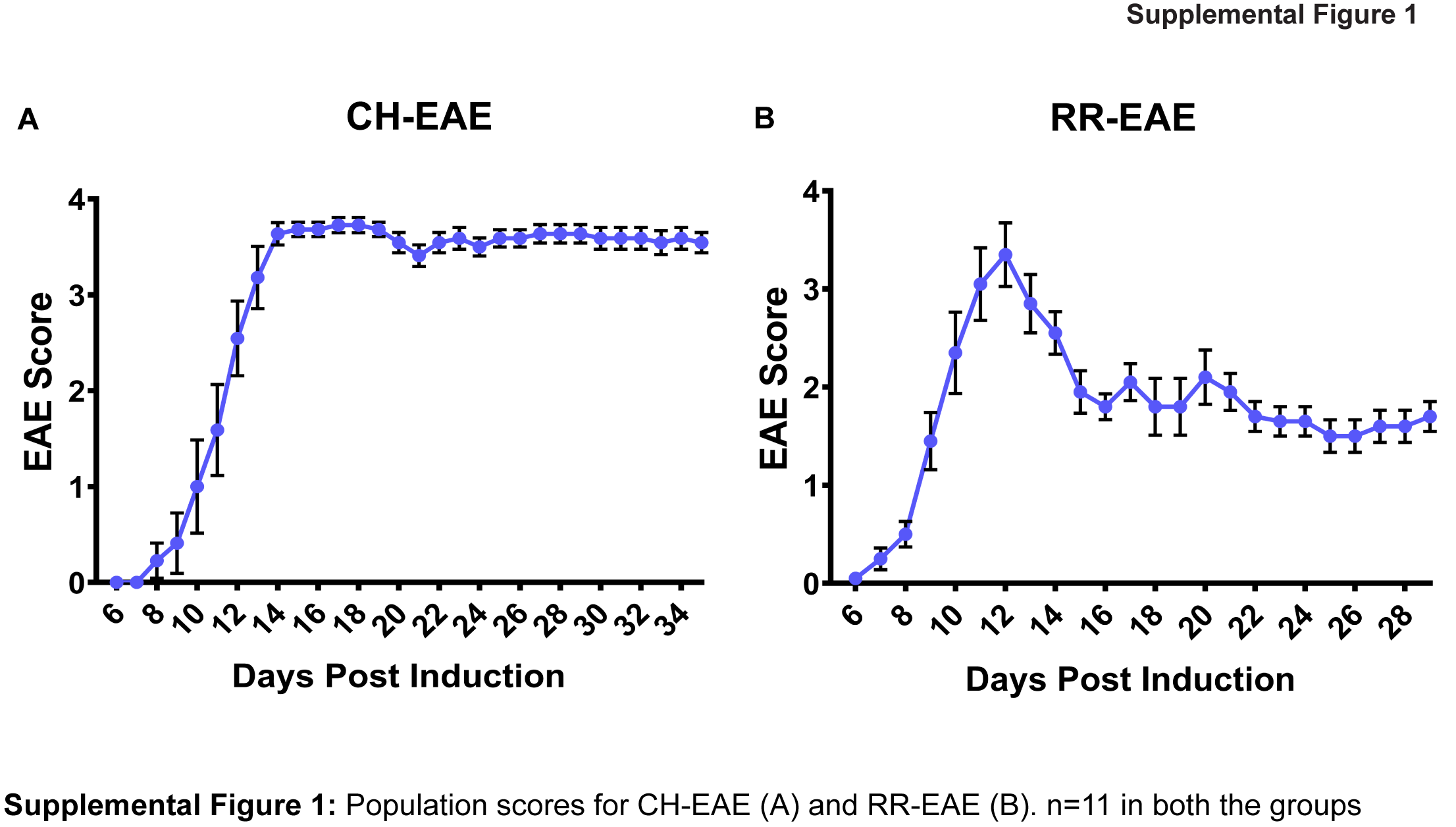

Supplement: Supplementary file 1 — Additional file 1: Figure 1. EAE population scores. [file 40478_2023_1617_MOESM1_ESM.tif]

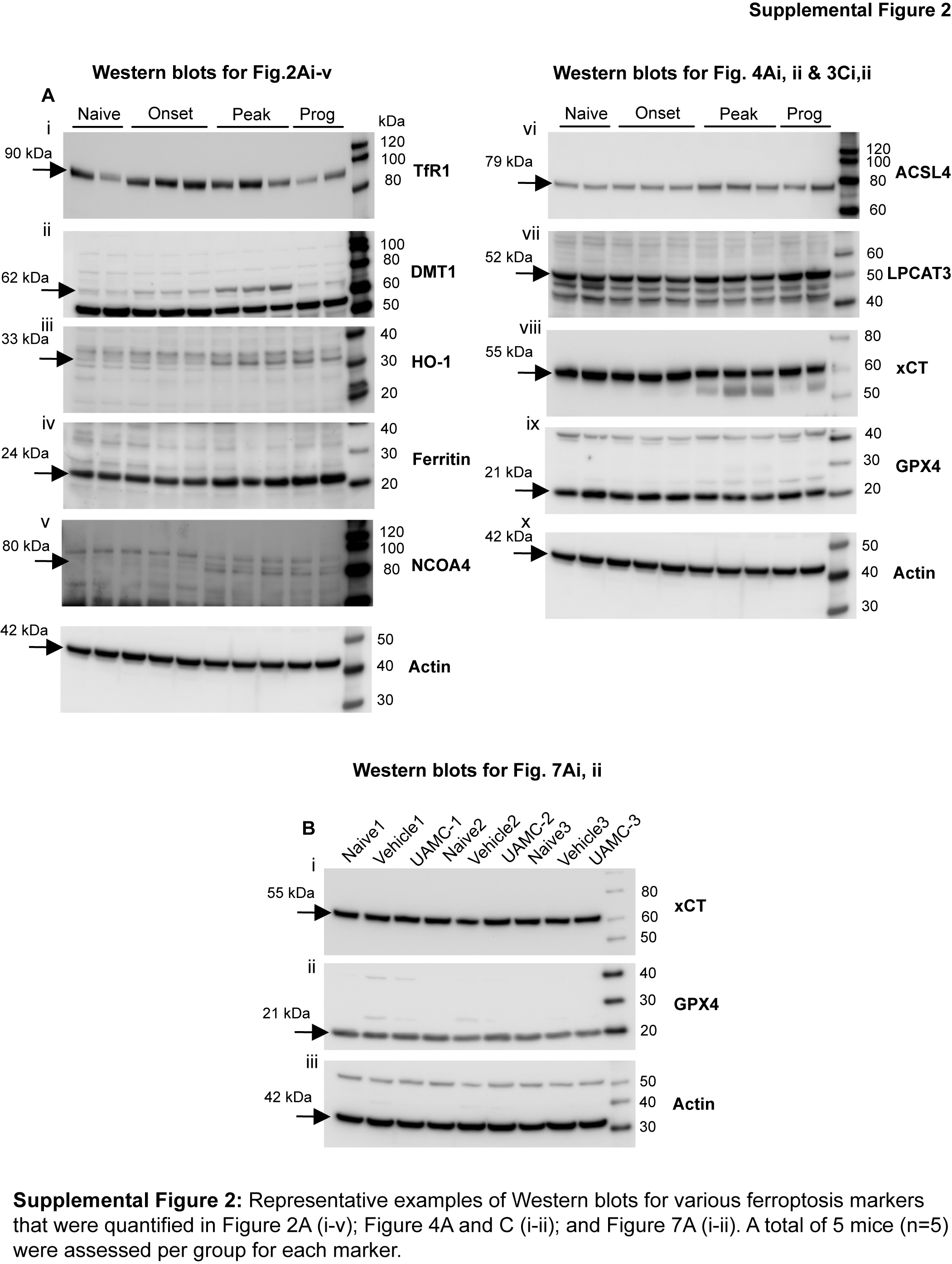

Supplement: Supplementary file 2 — Additional file 2: Figure 2. Western blots. [file 40478_2023_1617_MOESM2_ESM.tif]

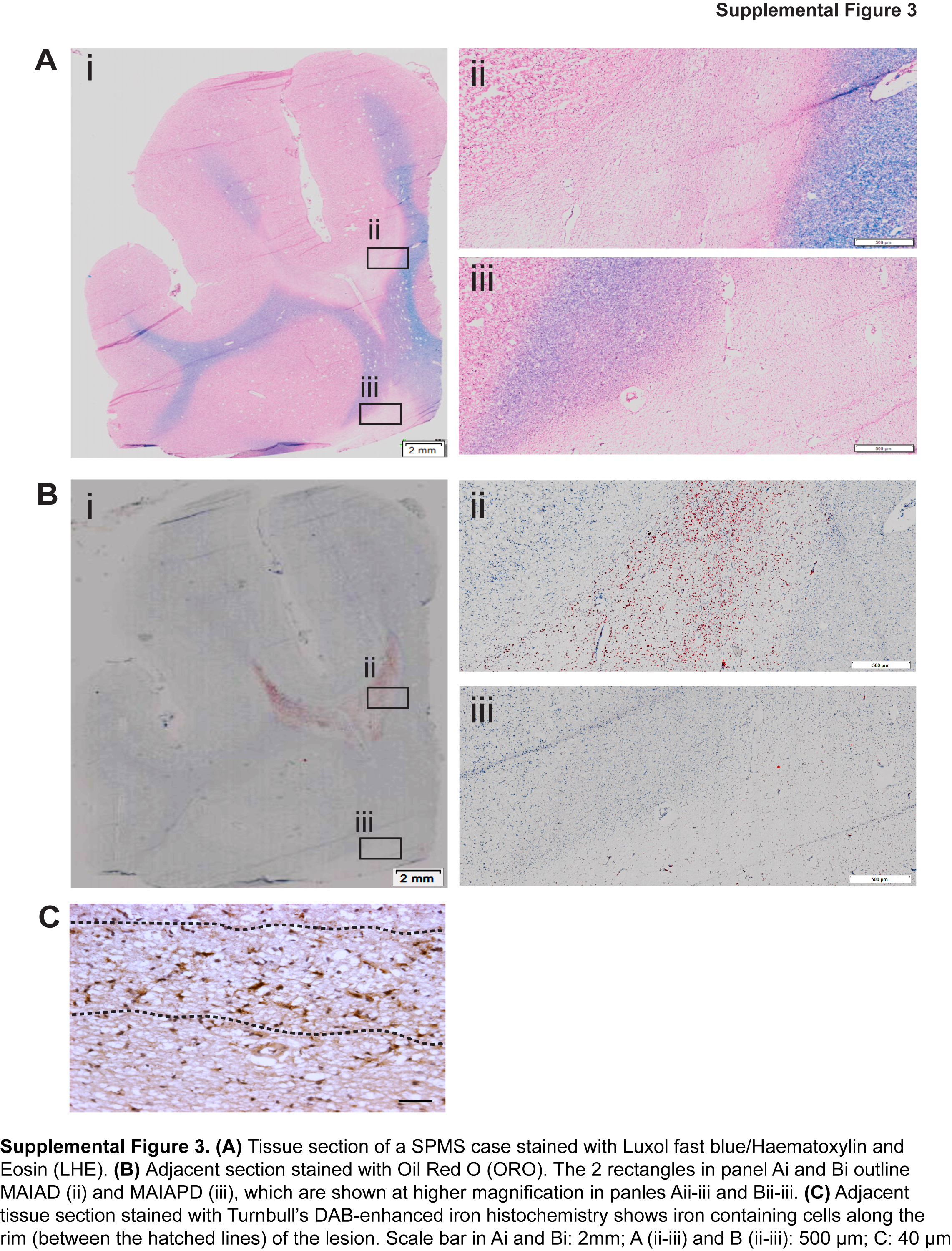

Supplement: Supplementary file 3 — Additional file 3: Figure 3. LHE-ORO staining and iron histochemistry of human MS tissue sections. [file 40478_2023_1617_MOESM3_ESM.tif]

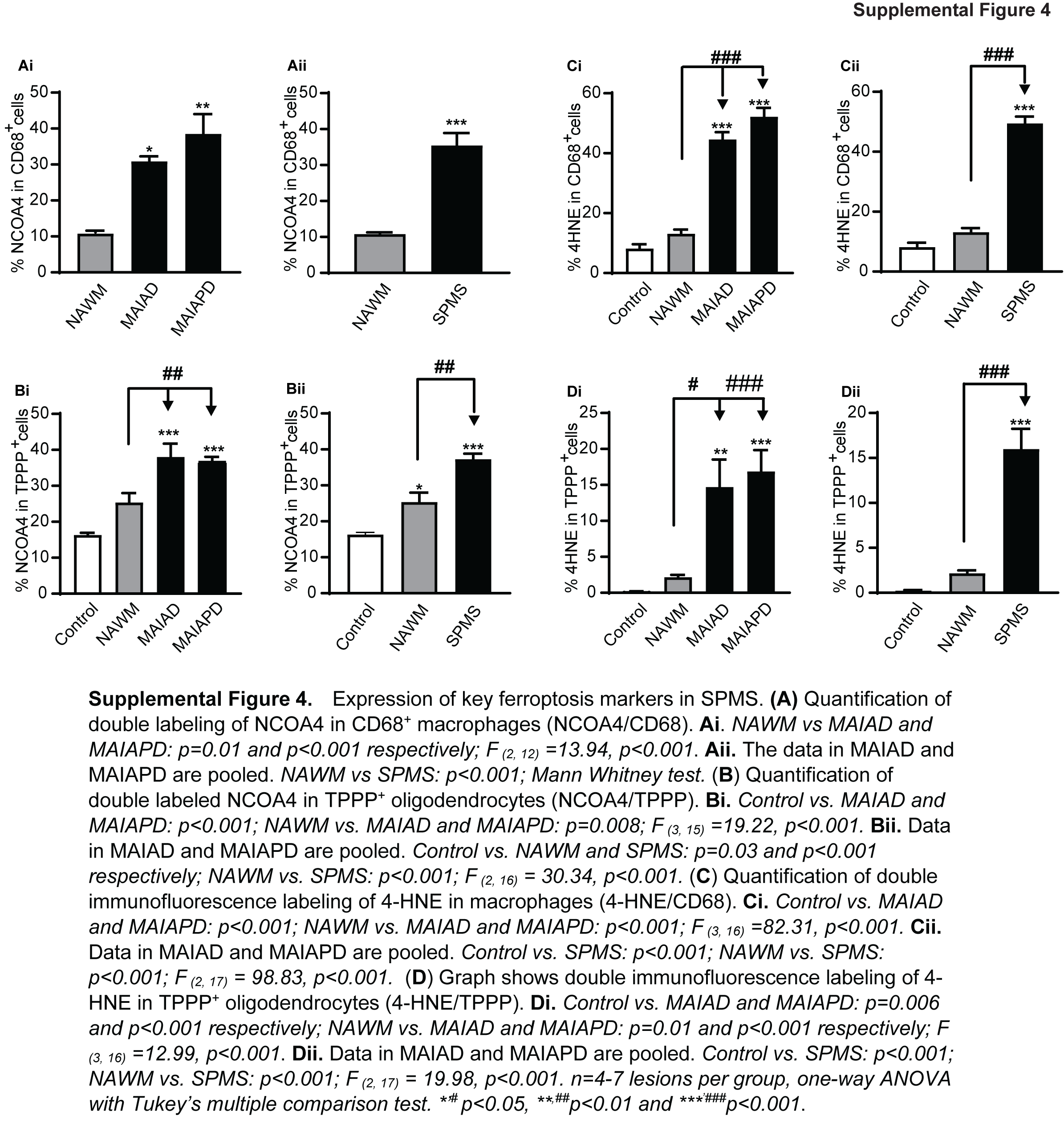

Supplement: Supplementary file 4 — Additional file 4: Figure 4. Quantification of double immunofluorescence labelled cells of Human tissue sections. [file 40478_2023_1617_MOESM4_ESM.tif]
