# Supplementary material for: Ferroptosis induces detrimental effects in chronic EAE and its implications for progressive MS
Source: Acta Neuropathol Commun. 2023 Jul 25;11:121. doi: 10.1186/s40478-023-01617-7 (PMC10369714; doi:10.1186/s40478-023-01617-7)
Supplement: Supplementary file 5 — Additional file 5: Methods. [file 40478_2023_1617_MOESM5_ESM.doc]

**Supplemental Methods**

Primer sequences used for Q-PCR

| Gene | Forward sequence | Reverse sequence |
| --- | --- | --- |
| *ireb2* | GGTGACCTACAGAAAGCAGGAA | TTCAGGTTCAGGCACTGGTT |
| *tfr1* | AAACTGGCTGAAACGGAGGAGACA | GCTGCTTGATGGTGTCAGCAAACT |
| *ncoa4* | AACACTGCCGACTGGGTTTT | AGCTGCATACAGGCAAAGAGA |
| *acsl4* | TGCTGCCTGTCCACTTGTTA | AGTATCTGCTCCAGGGATGTCT |
| *gpx4* | AAGTACAGGGGTTTCGTGTG | CGGCTGCAAACTCCTTGATT |
| *ppia* | ATGTGCCAGGGTGGTGACTTTA | TGTGTTTGGTCCAGCATTTGCC |

***Western blot:***

25µg of protein per sample was separated on 4%-12% SDS-PAGE gels (Novex, Invitrogen) and transferred to PVDF membrane (Millipore). Membranes were blocked with 5% milk in 0.05% PBS Tween-20 and probed with primary antibodies against TfR1, DMT1, HO-1, ferritin, NCOA4, ACSL4, LPCAT3, GPX4, and xCT. Details of antibodies provided below. Primary antibodies were incubated overnight at 4°C, blots were then washed and incubated with secondary antibodies: anti-mouse and anti-rabbit HRP-conjugated IgG (1:20,000-50,000; Jackson ImmunoResearch Laboratories) and the bands visualized by enhanced chemiluminescence using ChemiDoc MP (Bio-Rad). Equal loading of protein was assessed by re-probing blots with rabbit anti-β-actin antibody (1:1000; Sigma Millipore; A2066), and blots quantified using ImageJ software (version 1.54b, National Institutes of Health).

**List of antibodies used:**

Mouse anti-transferrin receptor (TfR1, 1:1000; Invitrogen; 13-6800)

Rabbit anti-DMT1 (1:1000; Alpha Diagnostics; NRAMP23-S)

Rabbit anti-HO-1 (1:1000; Abcam; ab68477)

Rabbit anti-ferritin (1:500; Sigma-Aldrich Inc.; F5012)

Mouse anti-NCOA4 (1:500; Abnova; H00008031-M04)

Rabbit anti-ACSL4 (1:1000; Abcam; ab155282)

Rabbit anti-LPCAT3 (1:500; Novus Biologicals; NBP3-04752)

Mouse anti-GPX4 (1:500; AlphaDiagnostic; MAB5457)

Rabbit anti-xCT (1:1000; Abcam; ab175186)

***Immunofluorescence (mouse tissues)***

Tissue sections were first incubated with blocking solution containing 0.3% Triton X-100 (Sigma Millipore), 2% ovalbumin (Sigma Millipore), and 5% normal goat/donkey serum (Jackson ImmunoResearch Laboratories) in 0.01M PBS for 2–3h at room temperature to block non-specific antibody binding. Sections were then incubated with primary antibodies overnight at 4°C against ferritin, HO-1, NCOA4, ACLS4, 4-HNE, GPX4 and double labeled with CC1 and Cd11b (details of the antibodies and suppliers provided below). Sections were washed and incubated with appropriate fluorescent-conjugated secondary antibodies: anti-rabbit AlexaFluor-488, anti-mouse AlexaFluor-568, and anti-rat AlexaFlour-568 (1:500 for all; Invitrogen). Slides were coverslipped in ProLong gold antifade mounting media containing DAPI (Invitrogen). Tissue sections were viewed with a confocal laser scanning microscope (FluoView FV1000, Olympus) and micrographs taken with the FV10-ASW 3.0 software (Olympus) and processed using ImageJ software.

Confocal images of 3 - 4 sections of the thoracic spinal cord (40x) were taken per animal (n = 4-5 mice per group) and the cells quantified from digital images using Image J software. Only CC1+ and CD11b+ cell bodies with DAPI labeled nuclei located in the parenchyma of the ventromedial white matter of the spinal cord away from the edge of the tissue were counted. The total number of CD11b+ or CC1+ cells in a given area was counted, as well as the number co-expressing different markers, which was used to calculate the mean and percentage of positively labeled cells. Graphs plotted using GraphPad prism.

**List of antibodies used** (**mouse tissues**):

Rabbit anti-ferritin (1:200; Sigma-Aldrich Inc.; F5012)

Rabbit anti-HO-1 (1: 200; Abcam ; ab68477)

Rabbit anti-NCOA4 (1:400; Invitrogen; PA5-96398)

Rabbit anti-ACSL4 (1: 400), Abcam; ab155282)

Rabbit anti-4-HNE, 1:100; Abcam; ab46545)

Rabbit anti-GPX4, (1:300; Abcam; ab125066),

Mouse anti-CC1 (for mature OLs; 1:300; Calbiochem Millipore; OP80)

Rat anti-CD11b (for macrophages/microglia; 1:200; AbD Serotec; MCA711)

Mouse anti-NCOA4 (1:200; Abnova; H00008031-M04)

***Immunofluorescence (human tissues)***

Sections were incubated with primary antibodies against ferritin, NCOA4, 4HNE; and double labeled with antibodies against CD68 and TPPP (details of antibodies are listed below). Appropriate fluorescent-conjugated secondary antibodies: anti-rabbit and anti-mouse AlexaFluor-488, anti-mouse AlexaFluor-568, and anti-rat AlexaFlour-568 (1:500 for all; Invitrogen) were used. Post quenching incubation was done using Lipofuscin autofluorescence quencher (Cat #23007; Biotium) according to the manufacturer instructions. Slides were cover-slipped in ProLong gold antifade mounting media containing DAPI (Invitrogen).

Images were captured using confocal laser scanning microscope (FluoView FV1000, Olympus) and micrographs were taken in a Z-stack series with the FV10-ASW 3.0 software (Olympus). For quantifications, cells were quantified from digital images using Image J software and the mean and percentage were calculated and graphs plotted using GraphPad prism.

**List of antibodies used (human tissues):**

Mouse anti-ferritin (1:400; Abcam; ab218400)

Rabbit anti-ferritin (1:200; Sigma-Aldrich Inc.; F5012)

Rabbit anti-NCOA4 (1:100; Abcam; ab222071)

Rabbit anti-4-HNE, (1:100; Abcam; ab46545),

Mouse anti-CD68 (for macrophage; 1:100; Abcam; ab955)

Rat anti-TPPP (anti-tubulin polymerization promoting protein); (for mature OLs; 1:300; Enzo Life Sciences; ALX-803-342-C100).

***Immunohistochemistry for 4HNE (mouse tissues)***

Endogenous peroxidase was blocked with 0.3% H_2_O_2_ in PBS for 30 min at room temperature. Sections were blocked for 3hrs in a blocking solution containing 0.3%TX-100, 2% Ovalbumin, 5%NGS with avidin D solution (Cat#SP2001; Vector laboratories) followed by incubation with primary rabbit anti-4HNE antibody (1:200; ab46545; Abcam) in a blocking solution with biotin (Cat#SP2001; Vector laboratories) for overnight. Sections were washed in 0.05% PBS-Tween-20 and then incubated with secondary goat anti-rabbit biotinylated (1:500; Vector Laboratories, BA-1000) followed by incubation with avidin peroxidase (1:100; #A-351; Sigma) for 1hr at room temperature. Labeling was visualized with diaminobenzidine-tetrahydrochloride using the ImmPACT DAB peroxidase substrate kit (Vector Laboratories, SK-4105) following the manufacturer’s instructions.

***DAB-enhanced Turnbull blue staining for iron (human tissues)****.*

Iron histochemistry was done as previously described (Zarruk et al) with minor modification. In brief, tissue sections were first post-fixed with 4% PFA for 30 min at RT followed by PBS washing. Sections were then incubated in 10% ammonium sulfide for 90 min at room temperature (RT), then washed with distilled water and incubated for 30 min in a solution of 20% potassium ferricyanide (Sigma-Aldrich) and 1% HCl. After washing in water, endogenous peroxidase was blocked for 60 min at RT in 99 ml methanol + 0.01 M NaN3 (65 mg) + 1 ml 30% H2O2. This was followed by washes in 0.1 M phosphate buffer and iron in the tissue visualized with 0.025% 3,3′ diaminobenzidine (DAB) with 0.005% H2O2 in 0.1 M phosphate buffer (20 min RT). The reaction was stopped with tap water and sections were counterstained with Mayers Hematoxylin solution (Sigma-Aldrich MHS16-500ML) and slides were coverslipped with Entellan mounting solution (Electron Microscopy Sciences). Sections were viewed using an Axioskop2 plus bright field microscope (Carl Zeiss) at 20x magnification, and images were captured using the Bioquant Life Sciences software.
